# Identifying potential circulating miRNA biomarkers for the diagnosis and prediction of ovarian cancer using machine-learning approach: application of Boruta

**DOI:** 10.3389/fdgth.2023.1187578

**Published:** 2023-08-09

**Authors:** Farzaneh Hamidi, Neda Gilani, Reza Arabi Belaghi, Hanif Yaghoobi, Esmaeil Babaei, Parvin Sarbakhsh, Jamileh Malakouti

**Affiliations:** ^1^ Department of Statistics and Epidemiology, Faculty of Health, Tabriz University of Medical Sciences, Tabriz, Iran; ^2^ Road Traffic Injury Research Center, Tabriz University of Medical Sciences, Tabriz, Iran; ^3^ Department of Mathematics, Applied Mathematics and Statistics, Uppsala University, Uppsala, Sweden; ^4^ Department of Statistics, Faculty of Mathematical Science, University of Tabriz, Tabriz, Iran; ^5^ Department of Energy and Technology, Swedish Agricultural University, Uppsala, Sweden; ^6^ Department of Biological Sciences, School of Natural Sciences, University of Tabriz, Tabriz, Iran; ^7^ Interfaculty Institute for Bioinformatics and Medical Informatics (IBMI), University of Tübingen, Tübingen, Germany; ^8^ Department of Midwifery, Faculty of Nursing and Midwifery, Tabriz University of Medical Science, Tabriz, Iran

**Keywords:** artificial intelligence, Boruta, biomarker, feature selection, Gene Expression Omnibus, ovarian cancer, oncology

## Abstract

**Introduction:**

In gynecologic oncology, ovarian cancer is a great clinical challenge. Because of the lack of typical symptoms and effective biomarkers for noninvasive screening, most patients develop advanced-stage ovarian cancer by the time of diagnosis. MicroRNAs (miRNAs) are a type of non-coding RNA molecule that has been linked to human cancers. Specifying diagnostic biomarkers to determine non-cancer and cancer samples is difficult.

**Methods:**

By using Boruta, a novel random forest-based feature selection in the machine-learning techniques, we aimed to identify biomarkers associated with ovarian cancer using cancerous and non-cancer samples from the Gene Expression Omnibus (GEO) database: GSE106817. In this study, we used two independent GEO data sets as external validation, including GSE113486 and GSE113740. We utilized five state-of-the-art machine-learning algorithms for classification: logistic regression, random forest, decision trees, artificial neural networks, and XGBoost.

**Results:**

Four models discovered in GSE113486 had an AUC of 100%, three in GSE113740 with AUC of over 94%, and four in GSE113486 with AUC of over 94%. We identified 10 miRNAs to distinguish ovarian cancer cases from normal controls: hsa-miR-1290, hsa-miR-1233-5p, hsa-miR-1914-5p, hsa-miR-1469, hsa-miR-4675, hsa-miR-1228-5p, hsa-miR-3184-5p, hsa-miR-6784-5p, hsa-miR-6800-5p, and hsa-miR-5100. Our findings suggest that miRNAs could be used as possible biomarkers for ovarian cancer screening, for possible intervention.

## 1. Introduction

Ovarian cancer is most often found in granulosa cells or germ cells, with epithelial histology accounting for more than 90% of all ovarian cancer. Epithelial ovarian cancer (EOC) (1) is a widespread gynecologic malignancy in industrialized and developing countries (2), with approximately 230,000 new cases and nearly 140,000 deaths per year (3). In 2020, the United States was expected to see 21,750 new cases and 13,940 deaths (4), while Europe experienced 29,000 deaths (5). According to the International Federation of Gynecology and Obstetrics (FIGO), only 30% of advanced-stage cancer patients live for nearly 5 years after receiving a primary-stage prognosis (6, 7). Only 19% of ovarian cancer patients are diagnosed at its early stage due to the absence of robust and minimally invasive methods at its early detection (8). Hence, advanced approaches for the early screening of ovarian cancer are necessary for proper medication and timely treatment. Regarding the genetic basis of cancer malignancy, microarray technology (9) has recently been one of the most widely used tools to evaluate the functions of genes in related patients. MicroRNAs (miRNAs) are short (18–25 nucleotides in length) non-coding RNAs that have emerged as important translational gene regulators in cancer cells (6). The screening models currently available are insufficient, and accurate non-invasive molecular biomarkers are urgently needed. Many studies have looked at the expression profiles of miRNAs in tissue and serum samples from ovarian cancer patients to identify appropriate biomarkers (10). Even though in many studies miRNAs are still insufficient for clinical applications that are due to large-scale non-validation and inconsistencies in the diagnosis of devices (11–13), it could expand a new screening strategy that can differentiate cancerous from non-cancerous women. In addition, the comprehensive characteristics of circulating miRNAs enable us to produce optimal diagnostic models for ovarian cancer (11–14).

### 1.1. Related works

MicroRNA molecules can act as an important tool for the detection of ovarian cancer. Chung et al. (15) reported let-7b, miR-26a, miR-132, and miR-145 as potential biomarkers in ovarian cancer patients. Among the results of Yuan et al.’s (16) study, has-miR-6784-5p, has-miR-6800-5p, and has-miR-5100 are indicating ovarian-associated cancer signature. Jeon et al. (17) reported that the serum and tissue miR-1290 was significantly elevated in patients with epithelial ovarian cancer compared with patients with benign ovarian neoplasm. Chen et al. (18) reported a total of 19 miRNAs, which were identified by random forest models, that were important in cancer diagnosis. In this study, the top five miRNAs with the highest frequency were chosen to be the biomarker candidates for cancer screening, which has-miR-3184-5p achieved a high rank. Yaghoobi et al. (19) proposed a method called EBST that has identified 11 serum miRNAs as potential biomarkers associated with ovarian cancer; among the miRNAs set, has-miR-1228-5p and has-miR-6784-5p were also reported. Zhang et al. (20) reported the four miRNA models that showed very strong performances with AUCs > 0.95 in the biliary tract, bladder, colorectal, esophageal, gastric, glioma, liver, ovarian, pancreatic, and prostate cancers. This study provides proof-of-concept data in demonstrating that the four miRNA (hsa-miR-5100, hsa-miR-1343-3p, hsa-miR-1290, and hsa-miR-4787-3p) model has the potential to be developed into a simple, inexpensive, and non-invasive blood test for the early detection of multiple cancers with high accuracy. Using statistical approaches, Hamidi et al. (21) identified 10 miRNAs regulated in ovarian serum cancer samples compared with non-cancer samples in the publicly available data set GSE106817: hsa-miR-5100, hsa-miR-6800-5p, hsa-miR-1233-5p, hsa-miR-4532, hsa-miR-4783-3p, hsa-miR-4787-3p, hsa-miR-1228-5p, hsa-miR-1290, hsa-miR-3184-5p, and hsa-miR-320b. However, the approach of the previous study (21) failed to take into account the non-linearity structure in big data; therefore, in this paper, we are implementing a new machine-learning variable selection approach called Boruta to address this problem. We will observe that the new miRNAs will be explored by the new method that has not been recognized in the traditional methods.

### 1.2. Novel contributions

It is important to note that the choice of feature selection (FS) method should be tailored to the specific characteristics of the data set and research question at hand. Gene expression data are the representation of non-linear interactions among genes (22). By computing analysis of these data, it is expected to gain knowledge of gene functions and disease mechanisms. Statistical methods can only identify linear patterns, while non-linear patterns of relationships remain hidden. As mentioned in many research (23–29), Boruta has superior advantages in terms of feature selection accuracy, stability, and classification performance across different domains such as protein subcellular localization and credit risk assessment, however, especially in microarray data sets of ovarian cancer that have been rarely used before. This is based on some studies on the stability of Boruta (30–32) as a machine-learning method that can more accurately discover new miRNAs that were hidden in statistical methods. Therefore, this work attempts an innovation in two important issues: the identification of new miRNAs based on complex non-linear structures and the comparison of new results with the previous ones, which will be described in the results and discussion section.

## 2. Materials and methods

To identify a robust circulating miRNA biomarker, we searched the Gene Expression Omnibus (GEO) database with specific keywords, namely, (“ovarian neoplasms” [MeSH Terms] OR ovarian cancer [All Fields]) AND “Homo sapiens” [porgn] AND “MicroRNAs” [MeSH Terms] OR miRNA [All Fields]. Then, three data sets using the same platform (3D-Gene Human miRNA V21_1.0.0) with a larger sample size GSE106817, GSE113486, and GSE113740 were included (385 ovarian cancer patients and 3,026 non-cancer controls in total) for further analysis. The GSE106817 has 320 ovarian cancer patients with an average age of 52 years and 2,759 non-cancer controls that were used as the internal discovery data set, and the GSE113486 has 40 ovarian cancer patients and 52 non-cancer controls. The GSE113740 has 25 ovarian cancer patients, and 215 non-cancer controls were used for independent validation data sets. This study was approved by the ethics committee of Tabriz University of Medical Sciences (no.: IR.TBZMED.REC.1400.006).

### 2.1. Study design and data set

We have used the GSE106817, GSE113486, and GSE113740 data sets from the GEO database, which is available at https://www.ncbi.nlm.nih.gov/geo/. The GSE106817 data set started on 13 November 2017 in Kanagawa, Japan, which is serum miRNA profiles of 4,046 women specimens, and which consists of 333 ovarian cancer and 2,759 non-cancer controls and 976 other types of cancer. The GSE106817 data set consists of ovarian cancer patients who were of mean age 57(±12) years, 25% stage I, 10% stage II, 55% serous, 19% clear cell, and 13% endometrioid histology (33). Three microarray data sets totaling to 6,835 unique participants including 728 ovarian cancer patients and 3,892 non-cancer controls were included in the current analysis, all derived from studies originating from a Japanese nationwide research project “Development and Diagnostic Technology for Detection of miRNA in Body Fluids” that is designed to characterize serum miRNAs in over 5,000 participants across several types of cancer using a standardized microarray platform. Supplementary Figure S1 clearly shows the stages of data pre-processing, identification of significant features or predictors, the model building of classifier algorithms, and performance evaluation, which are the four main phases of this analysis.

#### 2.1.1. Participants and serum samples

The serum sample collection has been previously described in the original publications (33–35). Briefly, serum samples were collected from cancer patients who were referred or admitted to the National Cancer Center Hospital (NCCH) and stored at 4°C for 1 week before being stored at −20°C until further use. Cancer patients who were treated with preoperative chemotherapy and radiotherapy before serum collection were excluded. The serum samples for non-cancer controls who had no history of cancer and no hospitalization during the previous 3 months were collected along with routine blood tests from outpatient departments of three sources: NCCH, National Center for Geriatrics and Gerontology (NCGG) Biobank, and Yokohama Minoru Clinic (YMC). Serums collected from NCCH were stored in the same way as the serum from cancer patients, while those from NCGG and YMC were stored at −80°C until use. The original studies were approved by the NCCH Institutional Review Board, the Ethics and Conflict of Interest Committee of the NCGG, and the Research Ethics Committee of Medical Corporation Shintokai YMC. Written informed consent was obtained from each participant.

#### 2.1.2. MiRNA microarray expression analysis

The details about microarray analysis were described in the original publications (33–35). Briefly, total RNA was extracted from a 300 µl serum, labeled by 3D-Gene® miRNA labeling kit and hybridized to 3D-Gene® Human miRNA Oligo Chip (Toray Industries, Kanagawa, Japan) that is designed to investigate 2,588 miRNA sequences registered in miRBase release 21 (http://www.mirbase.org/, accessed on 10 January 2022). The following low-quality samples were excluded: coefficient of variation of negative control probes of >0.15 and number of flagged probes identified by 3D-Gene® Scanner as “uneven spot images” of >10. The presence of a miRNA was determined when signal intensity was greater than the mean plus two times the standard deviation of the negative control signals, and in using the negative control signals, the top and bottom 5% of the ranked signal intensities were removed. Background subtraction was performed by subtracting the mean signal of negative control signals (after removing the top and bottom 5% as ranked by signal intensities) from the miRNA signal.

### 2.2. Machine learning

In cancer prediction models, statistical and machine-learning algorithms have been widely used, providing more accurate prognoses and lower per-patient costs. The high dimensionality of the gene expression profiles is a crucial issue when building cancer-predictive models (36). As a result, we used a machine-learning algorithm based on the random forest classifier, which is easily implemented in the Boruta package in R (37). In many studies involving miRNAs expression data, Boruta has been used to identify important features (38); this could help in the development of biomarkers for cancer diagnosis and prognosis. On the other hand, we used these techniques to characterize miRNAs with biomarker potential that may be useful in the diagnosis and/or prognosis of this disease, potentially assisting public health (39).

### 2.3. Data cleaning and feature selection

We cleaned and normalized the data using the min-max normalization method (40). Since gene expression data sets had too many irrelevant features for classification, feature selection was inevitable. Feature selection techniques can be used in data pre-processing to perform successful data reduction, which is beneficial for finding accurate data models (41). As noted, feature selection techniques have the benefits of reducing over-fitting and reducing model complexity with ease of understanding, as well as training models more quickly.

#### 2.3.1. Boruta

Boruta is a wrapper-based feature selection algorithm that implements a random forest algorithm to iteratively delete the statistically irrelevant features. Boruta searches for all features that are either strongly or weakly relevant to the output variable (27).

Boruta algorithm selects features as follows:

(a) It assigns randomness to the data set by making shuffled copies of all features (termed as shadow features).
(b) Next, Boruta uses the data set for training a random forest classifier and uses a feature ranking measure (mean decrease accuracy, MDA) to estimate the relationship with each feature (higher mean value).
(c) It determines whether a real feature has higher rank than the best of its shadow features on each iteration (in our analysis, 100) and excludes features that are considered extremely insignificant.
(d) Boruta algorithm comes to a halt when all features have been confirmed.

This would ultimately result in at least a subset of features that is ideal. Since this approach reduces the error of the random forest model, it identifies all features that are either highly significant or unrelated (32, 42, 43). Boruta is used in such a way that the features selected are mostly correlated with the prediction variable.

In the process of identifying if a feature is important or not, some features may be signed by Boruta as “Tentative.” Tentative attributes are decided as confirmed or rejected by using the median *Z* score of the attributes with the median *Z* score of the best shadow attribute.

### 2.4. Model building and potential miRNAs signature identification

We split the data using the CARET package into two parts: two-thirds of the data were used for model development or training, while the remaining one-third of the data were used to evaluate or validate the model.

#### 2.4.1. Handling of imbalanced classes

In most cases, prediction algorithms train to predict the majority class (i.e., non-cancer), resulting in incorrect sensitivities and specificities (44). Instead, fixing the imbalance in the outcomes (i.e., lower cancer rates) in the training data usually leads to the creation of a better prediction model and a better trade-off between sensitivity and specificity (45). Oversampling the minority class and under-sampling the majority class are the most effective strategy for overcoming imbalanced outcomes (46). To balance the training sample in this article, we used SMOTE random oversampling (47).

#### 2.4.2. Find optimal hyperparameters and proposed models

We used a five-fold cross-validation (CV) in the training data set to reduce training errors and obtained the optimal hyperparameters in machine-learning algorithms (48). We performed cancer classification using logistic regression, artificial neural network, decision trees, random forest, and XGBoost (49) algorithms, and to build our models, we applied the varImp() function for finding the most important feature (in our study >80% importance) from each of the proposed models. A brief description of classifiers and their settings are given below or in references therein.

##### 2.4.2.1. Logistic regression

Logistic regression (LR) is used when the answer of a feature is computed as numerical (quantitative) data. The relationship between multiple independent variables and a single binary dependent variable, which is a two-category variable, is investigated using logistic regression. In cancer microarray data, which is a form of the data set in which the outcome (cancer) is determined by the combined outcome of many features (genes), logistic regression has a variety of uses. Logistic regression rejects a linear relationship between the dependent and independent variables in favor of the binomial probability principle, which states that there are only two possible outcomes (50). The fit of a logistic regression model will be evaluated using the area under the curve (AUC) (51).

##### 2.4.2.2. Decision trees

Decision trees (DTs) are a type of supervised machine learning that can be used to find attributes and extract patterns in big databases that are important for predictive modeling (46). The interoperability of the rendered model is a feature of decision tree modeling that distinguishes it from other techniques of pattern recognition. The most straightforward algorithm for processing a visual representation of the relationship between independent and dependent variables is decision trees (52). DTs are easy to build, train, interpret, and explain. However, the variation in the decision trees, in some instances, can be improved using random forests as the outcomes of randomly generated decision trees to produce a more impressive model.

##### 2.4.2.3. Random forest

Random forest (RF) is a supervised ensemble learning algorithm that provides a single combination of prediction accuracy and model interoperability among general machine-learning technique (39). RFs are an instance of ensemble learning, in which a complex model was developed by combining numerous simple decision tree algorithms, due to lower variance than single decision trees. Random forest is a meta-classification approach that fits a number of sub-classifiers (DTs) on various subsets of a data set, and the averages from each decision tree are used to ameliorate the accuracy of classification, the superiorities of RF that they decrease the over-fitting, thus improving accuracy. Random forests can be used to rate the importance of variables in a regression or classification problem (53).

##### 2.4.2.4. Artificial neural networks

In medical research, artificial neural networks (ANNs) have been widely employed (54, 55). When there are complex and non-linear relationships between variables, such algorithms work well. In a word, ANN takes predictors as inputs and connects them to multiple hidden layer combinations with appropriate weights to predict the outcome. The analyst must intelligently choose the hidden layers and weights (56).

##### 2.4.2.5. XGBoosting

Extreme gradient boosting is abbreviated as XGBoost (XGB). XGB is a decision-tree-based ensemble machine-learning algorithm that employs a scalable gradient boosting technique (57). XGB is a scalable machine-learning system for tree boosting. The most significant component of the success of XGBoost is its scalability across all scenarios. XGB scalability is due to a number of major systems and algorithmic enhancements, parallel and distributed computing speed up learning, allowing for more rapid model exploration. XGB also allows data scientists to process by utilizing out-of-core processing (53).

### 2.5. Evaluation criteria

The validation technique is widely used to avoid over-fitting and to check the validity of the models. We evaluated our outcomes employing two external data sets, as shown in the Supplementary Figure S1. The metrics utilized to assess the results of the classification models are expressed below:

$$\mathrm{Accuracy}:\;\;\mathrm{ACC}=\frac{\mathrm{TP}+TN}{\mathrm{TP}+\mathrm{FP}+\mathrm{TN}+\mathrm{FN}},$$

$$\mathrm{Sensitivity}:\;\;\mathrm{SEN}=\frac{\mathrm{TP}}{\mathrm{TP}+\mathrm{FN}},$$

$$\mathrm{Specificity}:\;\;\mathrm{SPC}=\frac{\mathrm{TN}}{\mathrm{TN}+\mathrm{FP}},$$

$$\mathrm{Kappa}:\;\;k=\frac{\mathrm{Pr}(a)-\mathrm{Pr}(e)}{1-\mathrm{Pr}(e)}$$

where:

1. TP (true positive) is the number of people who suffer from “cancer” among those who were diagnosed with “cancer.”
2. FP (false positive) depicts the number of persons who are “cancerous” but were diagnosed as “non-cancerous.”
3. FN (false negative) is the number of people wrongly found to be “non-cancerous.”
4. TN (true negative) states the number of “non-cancerous” correctly.
5. Pr(a) represents the observed agreement, and Pr(e) represents the chance agreement.

We tested classifier reliability for multi-class data sets using Kappa values, which reflect the compromise among real and expected values (58); positive predictive value (PPV) and negative predictive value (NPV) were also obtained (59). The one-sided DeLong's test was used to calculate the power for the ROC curves, which was done using the R package “pROC” (60).

## 3. Result

The data have 2,568 variables. In this initial variable section stage by Boruta, 199 variables were selected in 29 min. The training set included 2,156 samples, while the testing set included 923 samples. The training set consisted of 1,932 non-cancerous samples and 224 cancerous samples. After balancing the training data, the non-cancerous and cancerous samples became 1,121 and 1,035, respectively. The data set with reduced features is classified using LR (statistical), DT and RF (tree-based), ANN, and XGB (machine learning) classifiers. After finding the more important features (in our study over 80%) as shown in Supplementary Table S1, we identified 10 potential miRNAs, has-miR-1290, has-miR-1233-5p, has-miR-1914-5p, has-miR-1469, has-miR-4675, has-miR-1228-5p, has-miR-3184-5p, has-miR-6784-5p, has-miR-6800-5p, and has-miR-5100, from the GSE106817 data sets and were defined as the candidate miRNAs for ovarian cancer diagnosis. In Supplementary Table S2, we reported the *t*-test table to compare cancer and non-cancerous samples, and all of these miRNAs had significant *P*-value. Using the 10 selected miRNAs, the final machine-learning models with optimal hyperparameters are presented in Table 1.

**Table 1:** Hyperparameters and predictive power of models for ovarian cancer classification.

Classifier	Hyperparameters	AUC^a^ (%)	Accuracy (%)	Sensitivity (%)	Specificity (%)	Negative predictive value (NPV) %	Positive predictive value (PPV) %
Logistic regression	Parameters^b^	99.77	100.0	100	100.0	100.0	100.0
Decision trees	Cp = 0.01014493^c^	98.30	91.30	97.41	97.10	88.10	94.0
Random forest	Mtry = 2^d^	100.0	96.74	99.55	100.0	94.55	100.0
Artificial neural network	Size = 3^e^ and decay = 0.1^f^	99.93	100.0	98.84	98.74	100.0	100.0
XGBoosting	nrounds = 50, max_depth = 2, eta = 0.4^g^ gamma = 0^h^ colsample_bytree^i^ = 0.6 min_child_weight^j^^ ^= 1 and subsample = 0.75^k^	99.99	98.91	99.28	100.0	100.0	98.11

^a^ The area under the receiver operating characteristic curve (maximum) was used to select the optimal model.

^b^ The formula for logistic regression for the prediction of ovarian cancer is $p={\left(1+e^{\begin{array}{c} -(\mathrm{10.463}-\mathrm{18.25}(\mathrm{has}.\mathrm{miR}\mathrm{.5100})-\mathrm{29.63}(\mathrm{has}.\mathrm{miR}\mathrm{.6800}\mathrm{.5}p)-9.30(\mathrm{has}.\mathrm{miR}.6784.5p)-\;7.38(\mathrm{has}.\mathrm{miR}.3184.5p)+2.702(\mathrm{has}.\mathrm{miR}.1228.5p)\\ +11.33(\mathrm{has}.\mathrm{miR}.4675)-8.19(\mathrm{has}.\mathrm{miR}.1469)+0(\mathrm{has}.\mathrm{miR}.1914.5p)+5.70(\mathrm{has}.\mathrm{miR}.1233.5p)+9.08(\mathrm{has}.\mathrm{miR}.1290))\; \end{array}}\right)}^{-1}\;.$

^c^ The complexity parameter (cp) is used to control the size of the decision tree and to select the optimal tree size. If the cost of adding an additional variable to the decision tree from the current node is above the value of the cp, then tree building does not continue.

^d^ mtry is the number of variables available for splitting at each tree node. In the random forests literature, this is referred to as the mtry parameter.

^e^ Size is the number of units in a hidden layer.

^f^ Decay is the regularization parameter used to avoid over-fitting.

^g^ max-depth is used to control over-fitting as higher depth will allow the model to learn relations very specific to a particular sample.

^h^ gamma A node is split only when the resulting split gives a positive reduction in the loss function. Gamma specifies the minimum loss reduction required to make a split, which makes the algorithm conservative. The values can vary depending on the loss function and should be tuned.

^i^ Denotes the fraction of columns to be randomly sampled for each tree.

^j^ min_child_weight is used to control over-fitting. Higher values prevent a model from learning relations that might be highly specific to the particular sample selected for a tree. Too high values can lead to under-fitting; hence, it should be tuned using CV.

^k^ subsample lower values make the algorithm more conservative and prevent over-fitting but too small values might lead to under-fitting.

### 3.1. Internal validation data set

**Figure 1:**
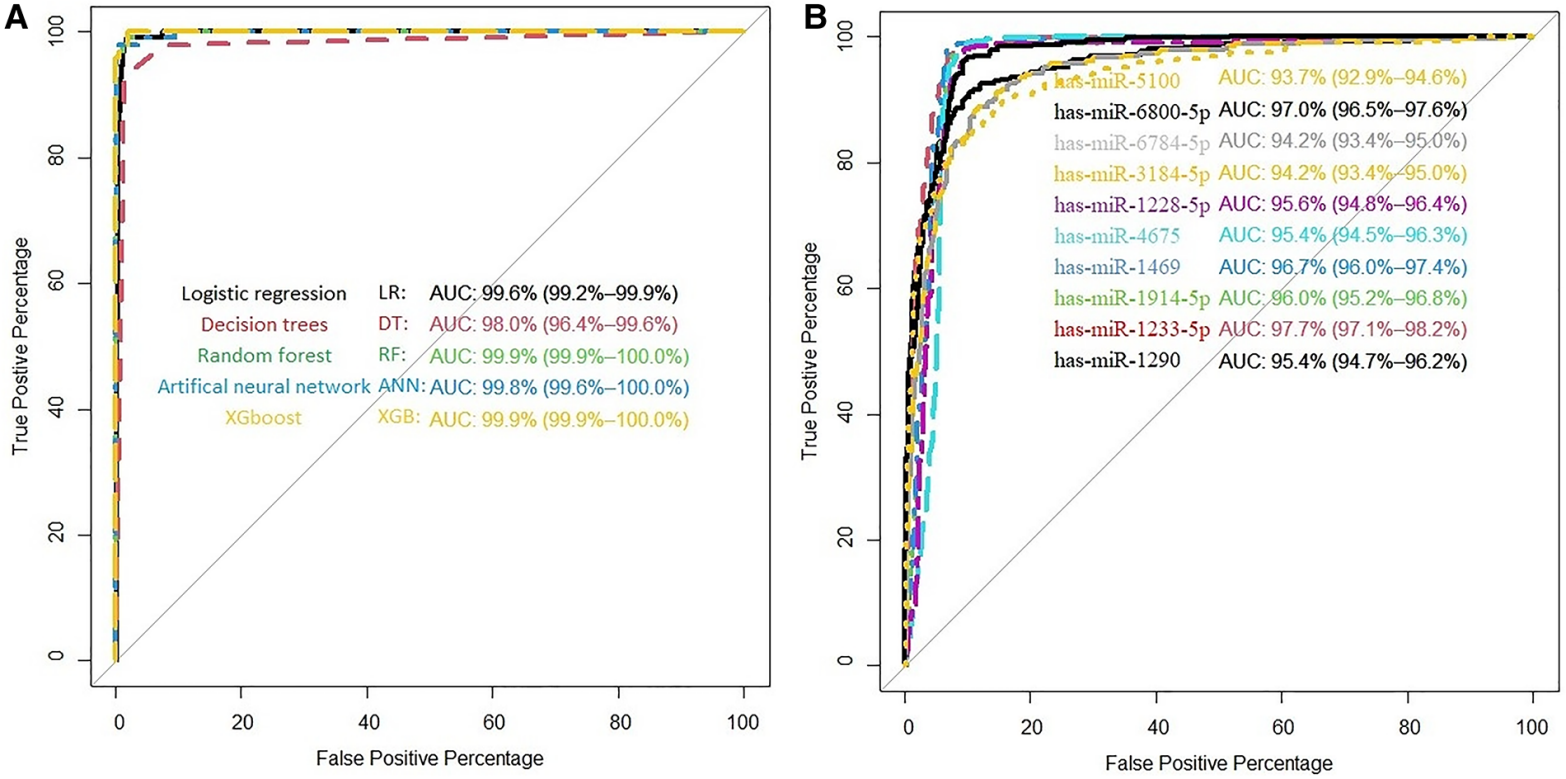
(**A**) ROC curve for the proposed models in GSE106817. (**B**) ROC curve of each selected miRNA in GSE106817.

As noted in the previous section, we find 10 miRNAs that are has-miR-1290, has-miR-1233-5p, has-miR-1914-5p, has-miR-1469, has-miR-4675, has-miR-1228-5p, has-miR-3184-5p, has-miR-6784-5p, has-miR-6800-5p, and has-miR-5100. We implemented each miRNA separately in models to get their power of prediction individually in classification between cancer and non-cancerous samples. The AUC of each of these miRNAs is listed in Supplementary Table S1A. We observe that in the internal validation, all miRNAs have high AUC (minimum AUC: 86.0%; maximum AUC is 96.8%). The performance measures for LR, DT, RF, ANN, and XGB models are shown in Supplementary Table S3A. We observe that the AUC of LR, RF, ANN, and XGB is 99.9%. Supplementary Table S3A shows the accuracy, sensitivity, specificity, NPV, PPV, and Kappa for LR, DT, RF, ANN, and XGB models in the classification and prediction of ovarian cancer. Four models obtained an AUC of 99.9%; however, DT obtained 98% AUC. In detail, RF has the highest value of accuracy (99.13), specificity (99.51), PPV (95.83), and Kappa (95.35), and LR have high sensitivity (98.96) and NPV (99.88). Figure 1A illustrates the ROC curve for the proposed models of 10 candidate miRNAs that are shown in Supplementary Table S1A. All models except DT have over 99.9% of AUC. Figure 1B shows the individual AUCs of 10 miRNAs in internal data set: has-mir-5100 (93.7%), has-mir-6800-5p (97%), has-mir-6784-5p (94.2%), has-mir-3184-5p (94.2%), has-mir-1228-5p (95.6%), has-mir-4675 (95.4%), has-mir-1469 (96.7%), has-mir-1914-5p (96%), has-mir-1233-5p (97.7%), and has-mir-1290(95.4%). In Supplementary Figure S2, we used a boxplot to display the expression levels of these 10 candidate miRNAs in the cancer and non-cancer groups. In the boxplots, it is clear that four of the miRNAs has-miR-1233-5p, has-miR-1914-5p, has-miR-4675, and has-miR-5100 have higher expression level with various cut-off for cancerous samples, and on average, four of them (has-miR-1228-5p, has-miR-3184-5p, has-miR-6784-5p, and has-miR-6800-5p) have lower expression level for cancerous samples. We used heatmap plots by implementing the “heatmaply” package to underpin the potential relationships between features and the hierarchical clustering analysis using the selected features to recognize different samples in the internal discovery data sets. Supplementary Figure S3 shows a promising result of the hierarchical clustering analysis (heatmap) using the 10 identified miRNAs to differentiate between cancerous and non-cancerous samples in GSE106817. The selected microRNAs are differently expressed in the non-cancer and cancerous classes. This is well illustrated by drawing the heatmap (Supplementary Figure S3).

### 3.2. External validation data sets

Supplementary Table S1B,C demonstrate the performance of miRNAs individually in proposed classification models in external validation data sets, as seen by the fact that almost all miRNAs have higher AUC in GSE113486 than in GSE113740. But in detail of each data set, Supplementary Table S3 shows the results of the external data set models based on the Boruta feature selection algorithm. As seen in Supplementary Table S3B LR and ANN have a value of 100 in all seven criteria and XGB has an AUC, specificity, and PPV of 100. Supplementary Figure S4A shows that all models for GSE113486 yielded 100% AUC except DT and RF. Supplementary Figure S4B illustrates how biomarkers perform individually has-mir-5100 (95.8%), has-mir-6800-5p (99.7%), has-mir-6784-5p (97.5%), has-mir-3184-5p (93.9%), has-mir-1228-5p (99.8%), has-mir-4675 (99.1%), has-mir-1469 (100%), has-mir-1914-5p (99.8%), has-mir-1233-5p (99.4%), and has-mir-1290 (96.4%) in GSE113486. Boxplots show us that six of miRNAs (has-mir-1290, has-mir-1233-5p, has-mir-1914-5p, has-mir-1469, has-mir-4675, and has-mir-5100) have upregulated to ovarian cancer samples in GSE113486 (Supplementary Figure S5). In GSE113740, as the second external validation data set, we can see the result of AUC of LR, RF, ANN, and XGB over 94% in Supplementary Figure S6A. We also found AUC for these 10 miRNAs (individually) in external data sets that included individually has-mir-5100 (90.6%), has-mir-6800-5p (89.7%), has-mir-6784-5p (74.4%), has-mir-3184-5p (74.4%), has-mir-1228-5p (85.2%), has-mir-4675 (79.7%), has-mir-1469 (84.4%), has-mir-1914-5p (81.5%), has-mir-1233-5p (86.5%), and has-mir-1290 (91%) as shown in Supplementary Figure S6B. Supplementary Table S3C shows us that RF and XGB have the highest value in Kappa (72.96 and 71.96) in AUC and accuracy (97.2, 93.75), as seen ANN has 100 of sensitivity and NPV. Boxplots (Supplementary Figure S7) show us that six of miRNAs (has-mir-1290, has-mir-1233-5p, has-mir-1914-5p, has-mir-1469, has-mir-4675, and has-mir-5100) have high expression level in ovarian cancer samples. Supplementary Figures S8, S9 show a promising result of the hierarchical clustering analysis (heatmap) using the 10 identified miRNAs to differentiate between the cancerous and non-cancerous samples in GSE113486 and GSE113740, respectively.

## 4. Discussion

It is critical to find and develop non-invasive, sensitive, and specific biomarkers to identify ovarian cancer in its early stages to effectively manage ovarian cancer patients. Fortunately, despite these limitations, newly discovered small RNAs called microRNAs have the potential to serve as effective non-invasive biomarkers for ovarian cancer (61, 62). Therefore, in this study, we used effective strategies and identified 10 miRNAs, hsa-miR-5100, hsa-miR-6800-5p, hsa-miR-6784-5p, hsa-miR-3184-5p, hsa-miR-1228-5p, hsa-miR-4675, hsa-miR-1469, hsa-miR-1914-5p, hsa-miR-1233-5p, and hsa-miR-1290, as strong potential biomarkers for ovarian cancer.

### 4.1. Biological insight

**Figure 2:**
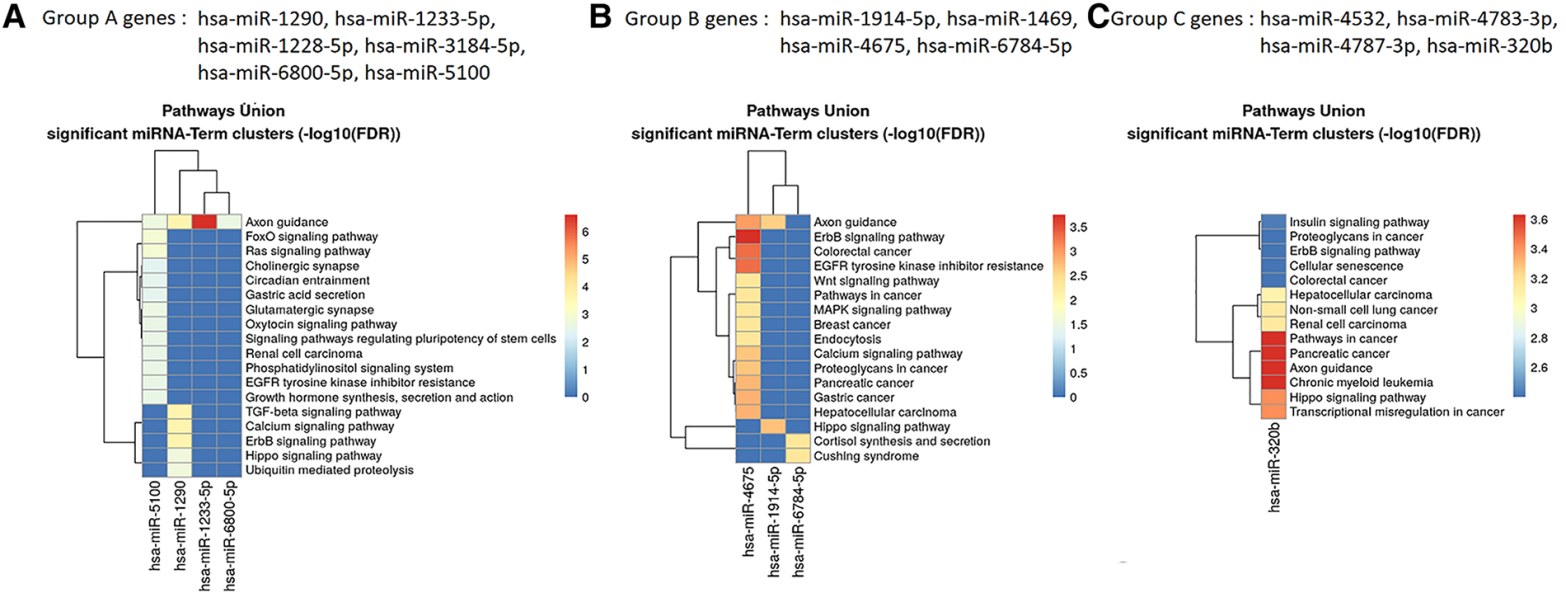
Targeted pathway clusters/heatmap presenting the top 10 Kyoto Encyclopedia of Genes and Genomes pathways regulated by the miRNAs (*P* < 0.005; DIANA/miRPath v.4).

**Figure 3:**
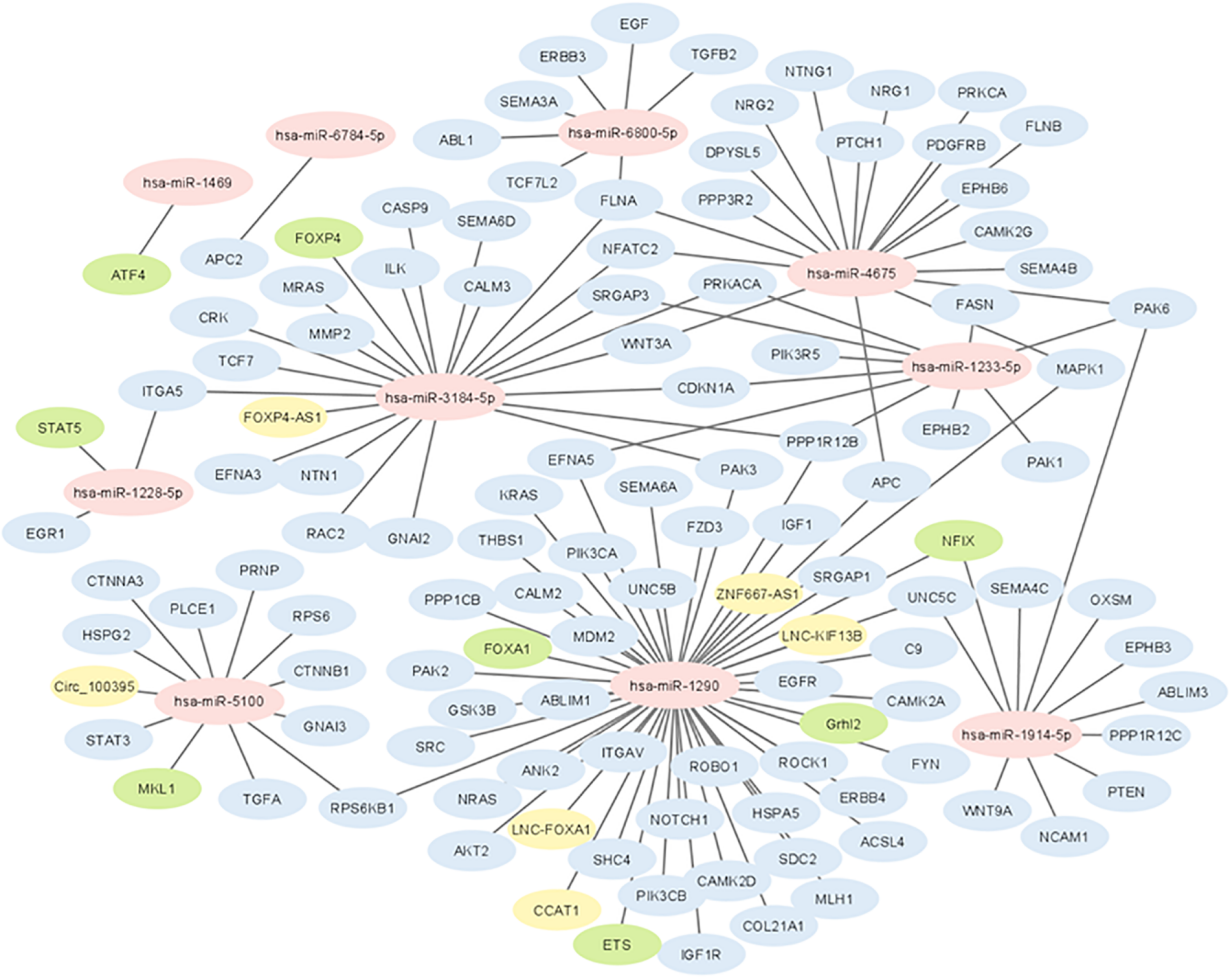
Network of interactions between selected miRNAs with coding genes and long non-coding RNAs. Yellow colored genes represent LNC-RNAs and green colored genes represent transcription factors.

The results of the biological insight section tell us about cell analysis for miRNAs that were found in this study based on the findings of the previous studies. The DIANA tool miRPath v.4 was used to perform the pathway enrichment analysis, based on the Kyoto Encyclopedia of Genes and Genomes (KEGG) database. The target genes of miRNA were identified using TargetScan v8.0 databases. The settings of the software were *P*-value threshold = 0.005 and the FDR correction filter were ticked. It should be mentioned that we used two methods to find the target genes: the first one is the genes union and the second is the pathway union. To investigate the efficiency of the set of biomarkers selected by Boruta and their superiority over the previous similar work done by Hamidi et al. (21), three groups of miRNAs were analyzed by miRPath v.4: (A) common biomarkers of the current study and the previous study by Hamidi et al. (21); (B) biomarkers selected by Boruta in the present study and not identified in the previous work; and (C) biomarkers that were selected in the previous study and were not identified in the current study. The list of genes of these three groups and their analysis results by miRPath v.4 tool are shown in Figure 2. As shown in Figure 2A, among the six common genes between the present and previous work, four genes are involved in at least one known cancer pathway (axon guidance). Among those four genes, hsa-miR-5100 and hsa-miR-1290 are involved in several well-known and important pathways in cancer. Figure 2B shows that among the four specific genes identified by the Boruta technique, three genes are involved in at least two well-known pathways in cancer, among which hsa-miR-4675 is involved in several pathways. However, in Figure 2C, among the four specific genes identified in the previous work of Hamidi et al. (21), only the hsa-miR-320b gene is involved in several important cancer pathways. It should be noted that there are six common paths between Groups A and B, while there are four common paths between A and C. This means that there are more correlation between genes of Group A and B than of Group A and C. This interpretation shows the biological superiority of Boruta's technique over the previous work. A comparison between routes of Group B and C also provides interesting results. Eight pathways are common between the two groups, which are proteoglycans in cancer, ErbB signaling, colorectal cancer, hepatocellular cancer, pathways in cancer, pancreatic cancer, axon guidance, and Hippo signaling. Axon guidance pathway is common among all the three groups. Many axon guidance molecules regulate cell migration and apoptosis in normal and tumorigenic tissues (63). Supplementary Table S4 shows the target genes of the selected microRNAs and the associated KEGG pathways from the genes union method, which indicates the significance of the relationship between the microRNAs and the corresponding pathways under the specified threshold values. Figure 3 shows the network of miRNAs and identified target genes. In this figure, transcription factors and LNC-RNAs have also been added through some studies. References for these interactions are described in Supplementary Table S4.

**Figure 4:**
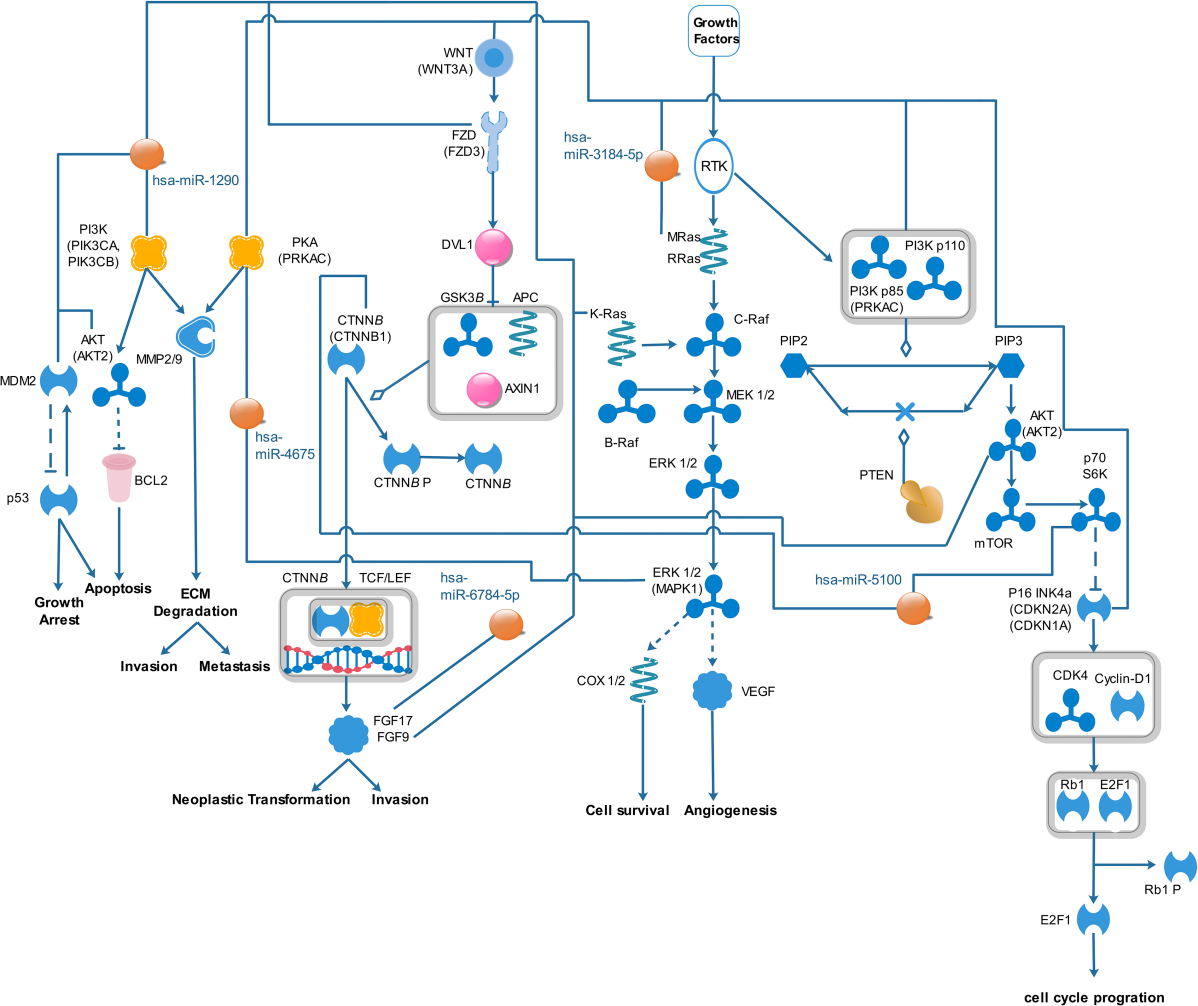
Predicted pathways of the effect of selected miRNAs in ovarian cancer.

In Supplementary Table S5, we only selected seven pathways because only the pathways that had very high correlation with miRNAs were selected (including a *P*-value of < 0.002). Among the top seven pathways identified, based on *P*-value, were pathways associated with fatty acid biosynthesis, prion diseases, axon guidance, glioma, ErbB signaling pathway, proteoglycans in cancer, and endometrial cancer. All signaling pathways related to miRNAs were used from known pathways, and in general, they play an important role in all types of cancer. According to the KEGG database, some of the published articles confirm the role of some of the selected miRNAs in cancer directly. A number of these documents are summarized in Table 2. Figure 4 shows the predicted pathways of the effect of some of the selected microRNAs that have been taken from the https://targetexplorer.ingenuity.com/index.htm.

**Table 2:** Summary of the role of selected miRNAs in cancer.

miRNA	Cancer type	Reference
hsa-miR-1290	Lung	Zhang et al. (64)
hsa-miR-1290	Colorectal	Imaoka et al. (65) Ye et al. (66)
hsa-miR-1290	Hepatocellular	Wang et al. (67)
hsa-miR-1290	Advanced oral squamous cell carcinoma	Nakashima et al. (68)
hsa-miR-1290	Pancreatic	Wei et al. (69)
hsa-miR-1290	Ovarian	Kobayashi et al. (70) Li et al. (71)
hsa-miR-1233-5p	Renal cell carcinoma	Dias et al. (72)
hsa-miR-1914-5p	Colorectal	Liu et al. (73)
hsa-miR-1914-5p	Epithelial ovarian	Chong et al. (74)
hsa-miR-1469	Pancreatic	Shams et al. (75)
hsa-miR-1469	Laryngeal	Ma et al. (76)
hsa-miR-1469	Colon	Gungormez et al. (77)
hsa-miR-4675	Breast	Lai et al. (78)
hsa-miR-4675	Various types	Chen and Dhahbi (18)
hsa-miR-3184-5p	Breast	Rajarajan et al. (79)
hsa-miR-3184-5p	Ovarian	Alshamrani (80)
hsa-miR-3184-5p	Various types	Chen and Dhahbi (18)
hsa-miR-6800-5p	Epithelial ovarian	Tuncer et al. (81)
hsa-miR-5100	Various types	Chen and Dhahbi (18)
hsa-miR-5100	Epithelial ovarian	Tuncer et al. (81)
hsa-miR-5100	Pancreatic	Chijiiwa et al. (82) Shams et al. (75)
hsa-miR-5100	Esophageal	Song et al. (83)
hsa-miR-1228-5p	Breast	Peña-Chilet et al. (84)
hsa-miR-1228-5p	Various types	Hu et al. (85)
hsa-miR-1228-5p	Breast	Cilek et al. (86)
hsa-miR-1228-5p	Hepatocellular	Morishita et al. (87)
hsa-miR-1228-5p	Epithelial ovarian	Chen et al. (88)
hsa-miR-1228-5p	Pancreatic	Wang et al. (89)
hsa-miR-6784-5p	Hepatocellular	Morishita et al. (87)
hsa-miR-6784-5p	Various types	Alshamrani (80)
hsa-miR-6784-5p	Esophageal	Song et al. (83)

**Figure 5:**
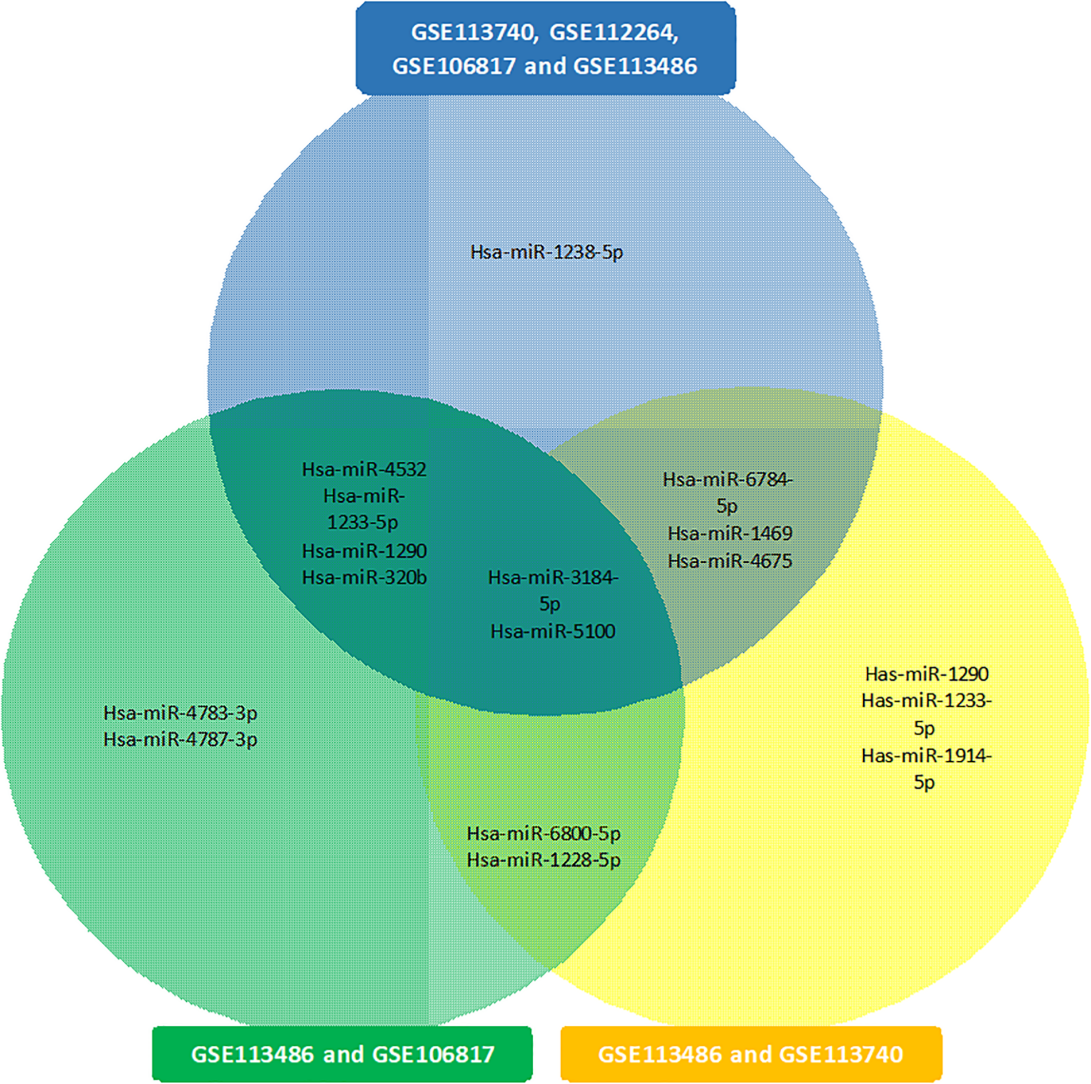
Venn diagram of common miRNAs among three different studies.

Figure 5 presents the common miRNAs between two related studies (18, 21) and miRNAs that were obtained in our study. There is some evidence in the literature for the biomarkers included in our study. Hamidi et al. (21) showed that hsa-miR-5100, hsa-miR-1233-5p, hsa-miR-4532, hsa-miR-1290, has-miR-3184-5p, and hsa-miR-320b could potentially be employed as important biomarkers in ovarian cancer. Jeon et al. (17) investigated that miRNA-1290 in the epithelial ovarian cancer group was significantly overexpressed in serum exosomes and tissues as compared with the benign ovarian neoplasm group. Ying et al. (90) expressed that microarray data analysis showed that hsa-miR-1290 was differentially expressed between COC1 (DDP-sensitive) and COC1/DDP (DDP-resistant) tumor cell lines. Chen et al. (18) showed that only five balanced miRNAs were determined to be important in cancer diagnosis: hsa-miR-663a, hsa-miR-6802-5p, hsa-miR-6784-5p, hsa-miR-3184-5p, and hsa-miR-8073. Furthermore, Chen et al. (18) found that hsa-miR-3184-5p can act as an early biomarker of bladder cancer and as a key regulator of breast cancer. Also, hsa-miR-6784-5p has been reported to be a sensitive serum biomarker for ovarian cancer diagnosis and a key regulator for breast cancer.

In the end, we note that although there are fundamental differences between microarray and RNA-Seq methods for obtaining gene expression data, the data matrix obtained from both methods is completely similar after performing the necessary pre-processing. Therefore, our method is also applicable to RNA-Seq data.

## 5. Strengths and limitations

This study provides several advantages. Firstly, to identify the relevant and important miRNAs, we utilized a robust variable selection method and a novel random forest-based feature selection of a machine-learning approach to identify and select the relevant and important miRNAs for ovarian cancer diagnosis, using Boruta as a novel random forest-based feature selection in the machine-learning techniques that has known roles in dimension reduction and select properties variables. Secondly, we used logistic regression and four of the most used machine-learning methods to predict and classify ovarian cancer. Thirdly, we selected three GEO data sets and ensured that they were from a similar platform, and used them in the evaluation stages. The first limitation of this study is that the biomarkers obtained in this study for ovarian cancer were not compared with the other common types of cancer in females. Secondly, the result of this study is possibly appropriate for a specific race or area because of the main data set.

## 6. Conclusion

Our study aimed to investigate reliable classification biomarkers in ovarian cancer. After utilizing Boruta for identifying the important biomarkers, we found 10 miRNAs that have high reliability in evaluating output from each classification model. The Hsa-miR-5100, hsa-miR-6800-5p, hsa-miR-6784-5p, hsa-miR-3184-5p, hsa-miR-1228-5p, hsa-miR-4675, hsa-miR-1469, hsa-miR-1914-5p, hsa-miR-1233-5p, and hsa-miR-1290 had significant differential expression in all models, especially in the two data sets studied (GSE106817, GSE113486). Except for decision trees, all the proposed models have performed fairly well in terms of the detection accuracy for ovarian cancer in the validation data sets. The LR, RF, ANN, and XGB in GSE106817 and GSE113486 data sets had over 99% AUC, and in GSE113740 over 94%. Even though this study presented some additional biomarkers for possible consideration in future research, the analyses in these data sets do not support the immediate clinical use of these biomarkers without more rigorous testing in large case-control and cohort studies.

## Data Availability

The original contributions presented in the study are included in the article/**Supplementary Material**; further inquiries can be directed to the corresponding author.
