# Supplementary material for: Identifying potential circulating miRNA biomarkers for the diagnosis and prediction of ovarian cancer using machine-learning approach: application of Boruta
Source: Front Digit Health. 2023 Aug 9;5:1187578. doi: 10.3389/fdgth.2023.1187578 (PMC10445490; doi:10.3389/fdgth.2023.1187578)
Supplement: Supplementary file 1 [file Datasheet1.docx]

Supplementary Table 1. AUC of 10 candidate miRNAs in 5 proposed predictive models.

| **a. Internal data GSE106817** | | | | | | |
| --- | --- | --- | --- | --- | --- | --- |
|  |  | AUC | | | | |
| No. | microRNAs | Logistic regression | Decision trees | Random forest | Artificial neural networks | XGBoost |
| 1 | has-mir-5100 | 95.9 | 88.4 | 91.4 | 95.9 | 95.5 |
| 2 | has-mir-6800-5p | 96.8 | 87.6 | 96.5 | 96.5 | 96.5 |
| 3 | has-mir-6784-5p | 94.2 | 92.3 | 92.6 | 95.0 | 94.1 |
| 4 | has-mir-3184-5p | 96.3 | 93.5 | 94.6 | 96.1 | 95.4 |
| 5 | has-mir-1228-5p | 94.9 | 94.2 | 94.0 | 96.2 | 95.8 |
| 6 | has-mir-4675 | 95.4 | 89.1 | 93.3 | 95.9 | 95.5 |
| 7 | has-mir-1469 | 94.2 | 86.2 | 89.0 | 94.2 | 94.1 |
| 8 | has-mir-1914-5p | 94.2 | 86.0 | 89.0 | 94.2 | 94.1 |
| 9 | has-mir-1233-5p | 96.8 | 94.3 | 95.1 | 96.8 | 96.3 |
| 10 | has-mir-1290 | 95.3 | 87.7 | 90.4 | 95.3 | 94.6 |
| **b. External data GSE113486** | | | | | | |
|  |  | AUC | | | | |
| No. | microRNAs | Logistic regression | Decision trees | Random forest | Artificial neural networks | XGBoost |
| 1 | has-mir-5100 | 99.4 | 96.7 | 95.6 | 99.4 | 96.7 |
| 2 | has-mir-6800-5p | 92.2 | 88.3 | 91.7 | 97.2 | 94.4 |
| 3 | has-mir-6784-5p | 100.0 | 100.0 | 100.0 | 100.0 | 100.0 |
| 4 | has-mir-3184-5p | 100.0 | 95.8 | 100.0 | 100.0 | 100.0 |
| 5 | has-mir-1228-5p | 100.0 | 95.8 | 95.6 | 100.0 | 100.0 |
| 6 | has-mir-4675 | 100.0 | 100.0 | 100.0 | 100.0 | 100.0 |
| 7 | has-mir-1469 | 99.4 | 86.7 | 90.0 | 99.4 | 98.3 |
| 8 | has-mir-1914-5p | 94.9 | 92.3 | 93.6 | 94.9 | 94.2 |
| 9 | has-mir-1233-5p | 100.0 | 96.2 | 100.0 | 100.0 | 96.2 |
| 10 | has-mir-1290 | 93.6 | 87.8 | 94.9 | 93.6 | 89.7 |
| **c. External data GSE113740** | | | | | | |
|  |  | AUC | | | | |
| No. | microRNAs | Logistic regression | Decision trees | Random forest | Artificial neural networks | XGBoost |
| 1 | has-mir-5100 | 89.7 | 90.5 | 93.1 | 91.4 | 95.4 |
| 2 | has-mir-6800-5p | 84.5 | 57.4 | 95.1 | 84.5 | 94.0 |
| 3 | has-mir-6784-5p | 77.6 | 84.5 | 85.1 | 83.6 | 86.5 |
| 4 | has-mir-3184-5p | 89.7 | 66.1 | 71.3 | 79.3 | 80.2 |
| 5 | has-mir-1228-5p | 83.0 | 85.9 | 95.7 | 95.7 | 97.4 |
| 6 | has-mir-4675 | 89.7 | 80.2 | 81.3 | 86.2 | 83.3 |
| 7 | has-mir-1469 | 63.2 | 54.9 | 48.3 | 63.2 | 52.0 |
| 8 | has-mir-1914-5p | 63.2 | 54.9 | 48.3 | 63.2 | 52.0 |
| 9 | has-mir-1233-5p | 97.1 | 82.8 | 86.2 | 93.7 | 87.9 |
| 10 | has-mir-1290 | 89.7 | 90.5 | 90.2 | 100 | 94.3 |

Supplementary Table 2. independent samples t-test for candidate miRNAs.

|  | **t-test for Equality of Means** | | | | |
| --- | --- | --- | --- | --- | --- |
|  | **Sig. (2-tailed)** | **Mean Difference** | **Std. Error Difference** | **95% Confidence Interval of the Difference** | |
|  |  |  |  | **Lower** | **Upper** |
| **hsa-miR-5100** | .000* | 2.79 | .06 | 2.66 | 2.92 |
| **hsa-miR-3184-5p** | .000* | -2.35 | .05 | -2.46 | -2.24 |
| **hsa-miR-6784-5p** | .000* | -1.19 | .02 | -1.25 | -1.1 |
| **hsa-miR-6800-5p** | .000* | -1.28 | .02 | -1.34 | -1.23 |
| **hsa-miR-1290** | .000* | 4.43 | .18 | 4.07 | 4.78 |
| **hsa-miR-1469** | .000* | 1.15 | .026 | 1.10 | 1.21 |
| **hsa-miR-1914-5p** | .000* | 1.90 | .067 | 1.77 | 2.038 |
| **hsa-miR-1233-5p** | .000* | 2.14 | .039 | 2.06 | 2.22 |
| **hsa-miR-4675** | .000* | 1.77 | .039 | 1.69 | 1.85 |
| **hsa-miR-1228-5p** | .000* | -.77 | .017 | -.80 | -.73 |

Supplementary Table 3. Predictive power of models for ovarian cancer classification and prediction in the internal and external validation datasets.

| **a. Internal data GSE106817** | | | | | | | |
| --- | --- | --- | --- | --- | --- | --- | --- |
| Classifier | AUC(%) | Accuracy(%) | Sensitivity (%) | Specificity (%) | Negative predictive value(%) | Positive  predictive value(%) | Kappa(%) |
| LR | 99.8 | 97.94 | 98.96 | 97.82 | 99.88 | 84.07 | 89.76 |
| DT | 97.5 | 97.94 | 92.70 | 98.54 | 99.14 | 88.11 | 89.20 |
| RF | 99.9 | 99.13 | 95.83 | 99.51 | 99.51 | 95.83 | 95.35 |
| ANN | 99.9 | 98.81 | 97.92 | 98.91 | 99.76 | 91.26 | 93.81 |
| XGB | 99.9 | 99.02 | 95.83 | 99.39 | 99.51 | 94.84 | 94.79 |
| **b. External data GSE113486** | | | | | | | |
| Classifier | AUC(%) | Accuracy(%) | Sensitivity (%) | Specificitiy(%) | Negative predictive value(%) | Positive  predictive value(%) | Kappa(%) |
| LR | 100.0 | 100.0 | 100.0 | 100.0 | 100.0 | 100.0 | 100.0 |
| DT | 92.6 | 91.30 | 92.50 | 90.38 | 94 | 88.10 | 82.4 |
| RF | 99.8 | 96.74 | 92.50 | 100.0 | 94.55 | 100.0 | 93.31 |
| ANN | 100.0 | 100.0 | 100.0 | 100.0 | 100.0 | 100.0 | 100.0 |
| XGB | 100.0 | 98.91 | 97.50 | 100.0 | 98.11 | 100.0 | 97.78 |
| **c. External data GSE113740** | | | | | | | |
| Classifier | AUC(%) | Accuracy(%) | Sensitivity (%) | Specificitiy(%) | Negative predictive value(%) | Positive  predictive value(%) | Kappa(%) |
| LR | 94.4 | 88.75 | 96.0 | 87.91 | 99.47 | 48.0 | 58.19 |
| DT | 88.9 | 85 | 92.0 | 84.18 | 98.90 | 10.31 | 48.66 |
| RF | 97.2 | 93.75 | 96.0 | 93.49 | 99.50 | 63.16 | 72.77 |
| ANN | 94.7 | 88.75 | 100.0 | 87.44 | 100.0 | 48.08 | 59.19 |
| XGB | 97.2 | 93.75 | 92.0 | 93.95 | 99.02 | 63.88 | 71.96 |

Supplementary Table 4. Transcription factors and long non-coding RNAs as targets of selected miRNAs.

| **Micro-RNA** | **Transcription Factor Target** | **Long Non-Coding RNA Target** | **References** |
| --- | --- | --- | --- |
| hsa-miR-1290 | FOXA1, Grhl2, NFIX, ETS | CCAT1, ZNF667-AS1, FOXA1, KIF13B | [1-3] |
| hsa-miR-1914-5p | NFIX | - | [4] |
| hsa-miR-1469 | ATF4 | - | [5] |
| hsa-miR-3184-5p | FOXP4 | LncRNA FOXP4-AS1 | [6, 7] |
| hsa-miR-5100 | MKL1 | - | [8] |
| hsa-miR-1228-5p | STAT5 | CircRNA hsa_circ_100395 | [9, 10] |

Supplementary Table 5. Top 10 KEGG pathways identified by miRNAs and genes targets.

| **KEGG pathway** | **p-value** | **miRNAs** | **genes** |
| --- | --- | --- | --- |
| **Fatty acid biosynthesis** **(hsa00061)** | **9.89E-19** | hsa-miR-1233-5p\|TargetScan\|microT-CDS | FASN |
|  |  | hsa-miR-1914-5p\|TargetScan\|microT-CDS | OXSM |
|  |  | hsa-miR-1290\|microT-CDS | ACSL4 |
| **Prion diseases** **(hsa05020)** | **4.97E-15** | hsa-miR-1290\|microT-CDS | FYN |
|  |  | hsa-miR-1290\|microT-CDS | HSPA5 |
|  |  | hsa-miR-1290\|microT-CDS | NOTCH1 |
|  |  | hsa-miR-1290\|microT-CDS | C9 |
|  |  | hsa-miR-1290\|microT-CDS | MAPK1 |
|  |  | hsa-miR-4675\|microT-CDS | MAPK1 |
|  |  | hsa-miR-1233-5p\|microT-CDS | PRKACA |
|  |  | hsa-miR-3184-5p\|TargetScan\|microT-CDS | PRKACA |
|  |  | hsa-miR-1914-5p\|TargetScan | NCAM1 |
|  |  | hsa-miR-5100\|microT-CDS | PRNP |
|  |  | hsa-miR-1228-5p\|Tarbase | EGR1 |
| **Axon guidance** **(hsa04360)** | **2.02E-05** | hsa-miR-1290\|microT-CDS | SEMA6A |
|  |  | hsa-miR-1290\|microT-CDS | GSK3B |
|  |  | hsa-miR-1290\|microT-CDS | NRAS |
|  |  | hsa-miR-1290\|microT-CDS | PAK2 |
|  |  | hsa-miR-1290\|microT-CDS | SRGAP1 |
|  |  | hsa-miR-1290\|microT-CDS | PAK3 |
|  |  | hsa-miR-1290\|microT-CDS | FYN |
|  |  | hsa-miR-1290\|microT-CDS | EFNA5 |
|  |  | hsa-miR-1290\|microT-CDS | UNC5C |
|  |  | hsa-miR-1290\|microT-CDS | UNC5B |
|  |  | hsa-miR-1290\|microT-CDS | ABLIM1 |
|  |  | hsa-miR-1290\|microT-CDS | ROBO1 |
|  |  | hsa-miR-1290\|microT-CDS | MAPK1 |
|  |  | hsa-miR-3184-5p\|microT-CDS\|TargetScan | RAC2 |
|  |  | hsa-miR-3184-5p\|microT-CDS | NTN1 |
|  |  | hsa-miR-3184-5p\|microT-CDS | EFNA3 |
|  |  | hsa-miR-3184-5p\|microT-CDS | PAK3 |
|  |  | hsa-miR-3184-5p\|microT-CDS | SRGAP3 |
|  |  | hsa-miR-3184-5p\|microT-CDS\|TargetScan | NFATC2 |
|  |  | hsa-miR-3184-5p\|microT-CDS\|TargetScan | GNAI2 |
|  |  | hsa-miR-3184-5p\|microT-CDS | SEMA6D |
|  |  | hsa-miR-4675\|microT-CDS\|TargetScan | NTNG1 |
|  |  | hsa-miR-4675\|microT-CDS | DPYSL5 |
|  |  | hsa-miR-4675\|microT-CDS\|TargetScan | NFATC2 |
|  |  | hsa-miR-4675\|microT-CDS\|TargetScan | SEMA4B |
|  |  | hsa-miR-4675\|microT-CDS | PAK6 |
|  |  | hsa-miR-4675\|microT-CDS | EPHB6 |
|  |  | hsa-miR-4675\|microT-CDS | MAPK1 |
|  |  | hsa-miR-4675\|microT-CDS\|TargetScan | PPP3R2 |
|  |  | hsa-miR-1233-5p\|microT-CDS | EPHB2 |
|  |  | hsa-miR-1233-5p\|microT-CDS | EFNA5 |
|  |  | hsa-miR-1233-5p\|microT-CDS\|TargetScan | PAK1 |
|  |  | hsa-miR-1233-5p\|microT-CDS | SRGAP3 |
|  |  | hsa-miR-1233-5p\|microT-CDS | PAK6 |
|  |  | hsa-miR-1914-5p\|microT-CDS | ABLIM3 |
|  |  | hsa-miR-1914-5p\|microT-CDS | EPHB3 |
|  |  | hsa-miR-1914-5p\|microT-CDS | UNC5C |
|  |  | hsa-miR-1914-5p\|microT-CDS | SEMA4C |
|  |  | hsa-miR-1914-5p\|microT-CDS | PAK6 |
|  |  | hsa-miR-1914-5p\|microT-CDS | PAK6 |
|  |  | hsa-miR-1290\|Tarbase | ROCK1 |
|  |  | hsa-miR-1290\|Tarbase | KRAS |
|  |  | hsa-miR-6800-5p\|microT-CDS | SEMA3A |
|  |  | hsa-miR-6800-5p\|microT-CDS | ABL1 |
|  |  | hsa-miR-5100\|Tarbase | GNAI3 |
| **Glioma** **(hsa05214)** | **0.000112173** | hsa-miR-1290\|Tarbase | KRAS |
|  |  | hsa-miR-1290\|Tarbase | CALM2 |
|  |  | hsa-miR-1290\|Tarbase | AKT2 |
|  |  | hsa-miR-1290\|Tarbase | MDM2 |
|  |  | hsa-miR-1290\|microT-CDS | CAMK2D |
|  |  | hsa-miR-1290\|microT-CDS | NRAS |
|  |  | hsa-miR-1290\|microT-CDS | PIK3CB |
|  |  | hsa-miR-1290\|microT-CDS | IGF1R |
|  |  | hsa-miR-1290\|microT-CDS | EGFR |
|  |  | hsa-miR-1290\|microT-CDS | CALM2 |
|  |  | hsa-miR-1290\|microT-CDS | CAMK2A |
|  |  | hsa-miR-1290\|microT-CDS | IGF1 |
|  |  | hsa-miR-1290\|microT-CDS | SHC4 |
|  |  | hsa-miR-1290\|microT-CDS | PIK3CA |
|  |  | hsa-miR-1290\|microT-CDS | MAPK1 |
|  |  | hsa-miR-4675\|microT-CDS | PRKCA |
|  |  | hsa-miR-4675\|microT-CDS | CAMK2G |
|  |  | hsa-miR-4675\|microT-CDS | MAPK1 |
|  |  | hsa-miR-4675\|microT-CDS | PDGFRB |
|  |  | hsa-miR-1233-5p\|microT-CDS | PIK3R5 |
|  |  | hsa-miR-1233-5p\|microT-CDS | CDKN1A |
|  |  | hsa-miR-3184-5p\|microT-CDS | CALM3 |
|  |  | hsa-miR-3184-5p\|microT-CDS | CDKN1A |
|  |  | hsa-miR-6800-5p\|microT-CDS | EGF |
|  |  | hsa-miR-5100\|microT-CDS | TGFA |
|  |  | hsa-miR-1914-5p\|microT-CDS | PTEN |
| **ErbB signaling pathway** **(hsa04012)** | **0.000365865** | hsa-miR-1290\|microT-CDS | CAMK2D |
|  |  | hsa-miR-1290\|microT-CDS | GSK3B |
|  |  | hsa-miR-1290\|microT-CDS | NRAS |
|  |  | hsa-miR-1290\|microT-CDS | PIK3CB |
|  |  | hsa-miR-1290\|microT-CDS | PAK2 |
|  |  | hsa-miR-1290\|microT-CDS | EGFR |
|  |  | hsa-miR-1290\|microT-CDS | PAK3 |
|  |  | hsa-miR-1290\|microT-CDS | CAMK2A |
|  |  | hsa-miR-1290\|microT-CDS | SRC |
|  |  | hsa-miR-1290\|microT-CDS | SHC4 |
|  |  | hsa-miR-1290\|microT-CDS | PIK3CA |
|  |  | hsa-miR-1290\|microT-CDS | MAPK1 |
|  |  | hsa-miR-1290\|microT-CDS | ERBB4 |
|  |  | hsa-miR-1290\|microT-CDS | RPS6KB1 |
|  |  | hsa-miR-1233-5p\|microT-CDS | PIK3R5 |
|  |  | hsa-miR-1233-5p\|microT-CDS | PAK1 |
|  |  | hsa-miR-1233-5p\|microT-CDS | PAK6 |
|  |  | hsa-miR-1233-5p\|microT-CDS | CDKN1A |
|  |  | hsa-miR-3184-5p\|microT-CDS | CRK |
|  |  | hsa-miR-3184-5p\|microT-CDS | PAK3 |
|  |  | hsa-miR-3184-5p\|microT-CDS | CDKN1A |
|  |  | hsa-miR-4675\|microT-CDS | PRKCA |
|  |  | hsa-miR-4675\|microT-CDS | CAMK2G |
|  |  | hsa-miR-4675\|microT-CDS | NRG2 |
|  |  | hsa-miR-4675\|microT-CDS | NRG1 |
|  |  | hsa-miR-4675\|microT-CDS | PAK6 |
|  |  | hsa-miR-4675\|microT-CDS | MAPK1 |
|  |  | hsa-miR-6800-5p\|microT-CDS | ERBB3 |
|  |  | hsa-miR-6800-5p\|microT-CDS | ABL1 |
|  |  | hsa-miR-6800-5p\|microT-CDS | EGF |
|  |  | hsa-miR-5100\|microT-CDS | TGFA |
|  |  | hsa-miR-5100\|microT-CDS | RPS6KB1 |
|  |  | hsa-miR-1290\|Tarbase | KRAS |
|  |  | hsa-miR-1290\|Tarbase | AKT2 |
|  |  | hsa-miR-1914-5p\|microT-CDS | PAK6 |
|  |  | hsa-miR-1914-5p\|microT-CDS | PAK6 |
| **Proteoglycans in cancer (hsa05205)** | **0.001316929** | hsa-miR-1290\|microT-CDS | CAMK2D |
|  |  | hsa-miR-1290\|microT-CDS | NRAS |
|  |  | hsa-miR-1290\|microT-CDS | THBS1 |
|  |  | hsa-miR-1290\|microT-CDS | PIK3CB |
|  |  | hsa-miR-1290\|microT-CDS | IGF1R |
|  |  | hsa-miR-1290\|microT-CDS | EGFR |
|  |  | hsa-miR-1290\|microT-CDS | PPP1R12B |
|  |  | hsa-miR-1290\|microT-CDS | FZD3 |
|  |  | hsa-miR-1290\|microT-CDS | ANK2 |
|  |  | hsa-miR-1290\|microT-CDS | COL21A1 |
|  |  | hsa-miR-1290\|microT-CDS | CAMK2A |
|  |  | hsa-miR-1290\|microT-CDS | ITGAV |
|  |  | hsa-miR-1290\|microT-CDS | SRC |
|  |  | hsa-miR-1290\|microT-CDS | IGF1 |
|  |  | hsa-miR-1290\|microT-CDS | PIK3CA |
|  |  | hsa-miR-1290\|microT-CDS | SDC2 |
|  |  | hsa-miR-1290\|microT-CDS | MAPK1 |
|  |  | hsa-miR-1290\|microT-CDS | ERBB4 |
|  |  | hsa-miR-1290\|microT-CDS | RPS6KB1 |
|  |  | hsa-miR-1290\|microT-CDS | PPP1CB |
|  |  | hsa-miR-1233-5p\|microT-CDS | PIK3R5 |
|  |  | hsa-miR-1233-5p\|microT-CDS | PPP1R12B |
|  |  | hsa-miR-1233-5p\|microT-CDS | PAK1 |
|  |  | hsa-miR-1233-5p\|microT-CDS | PRKACA |
|  |  | hsa-miR-1233-5p\|microT-CDS | CDKN1A |
|  |  | hsa-miR-3184-5p\|microT-CDS | ITGA5 |
|  |  | hsa-miR-3184-5p\|microT-CDS | MRAS |
|  |  | hsa-miR-3184-5p\|microT-CDS | PPP1R12B |
|  |  | hsa-miR-3184-5p\|microT-CDS | MMP2 |
|  |  | hsa-miR-3184-5p\|microT-CDS | PRKACA |
|  |  | hsa-miR-3184-5p\|microT-CDS | FLNA |
|  |  | hsa-miR-3184-5p\|microT-CDS | WNT3A |
|  |  | hsa-miR-3184-5p\|microT-CDS | CDKN1A |
|  |  | hsa-miR-1290\|Tarbase | ROCK1 |
|  |  | hsa-miR-1290\|Tarbase | KRAS |
|  |  | hsa-miR-1290\|Tarbase | AKT2 |
|  |  | hsa-miR-1290\|Tarbase | MDM2 |
|  |  | hsa-miR-4675\|microT-CDS | PRKCA |
|  |  | hsa-miR-4675\|microT-CDS | CAMK2G |
|  |  | hsa-miR-4675\|microT-CDS | PTCH1 |
|  |  | hsa-miR-4675\|microT-CDS | FLNB |
|  |  | hsa-miR-4675\|microT-CDS | FLNA |
|  |  | hsa-miR-4675\|microT-CDS | WNT3A |
|  |  | hsa-miR-4675\|microT-CDS | MAPK1 |
|  |  | hsa-miR-6800-5p\|microT-CDS | ERBB3 |
|  |  | hsa-miR-6800-5p\|microT-CDS | FLNA |
|  |  | hsa-miR-6800-5p\|microT-CDS | TGFB2 |
|  |  | hsa-miR-5100\|Tarbase | RPS6 |
|  |  | hsa-miR-5100\|Tarbase | CTNNB1 |
|  |  | hsa-miR-1228-5p\|Tarbase | ITGA5 |
|  |  | hsa-miR-5100\|microT-CDS | STAT3 |
|  |  | hsa-miR-5100\|microT-CDS | HSPG2 |
|  |  | hsa-miR-5100\|microT-CDS | PLCE1 |
|  |  | hsa-miR-5100\|microT-CDS | RPS6KB1 |
|  |  | hsa-miR-1914-5p\|microT-CDS | PPP1R12C |
|  |  | hsa-miR-1914-5p\|microT-CDS | WNT9A |
| **Endometrial cancer** **(hsa05213)** | **0.001479125** | hsa-miR-1914-5p\|microT-CDS | PTEN |
|  |  | hsa-miR-1290\|microT-CDS | GSK3B |
|  |  | hsa-miR-1290\|microT-CDS | NRAS |
|  |  | hsa-miR-1290\|microT-CDS | APC |
|  |  | hsa-miR-1290\|microT-CDS | PIK3CB |
|  |  | hsa-miR-1290\|microT-CDS | EGFR |
|  |  | hsa-miR-1290\|microT-CDS | MLH1 |
|  |  | hsa-miR-1290\|microT-CDS | PIK3CA |
|  |  | hsa-miR-1290\|microT-CDS | MAPK1 |
|  |  | hsa-miR-3184-5p\|microT-CDS | CASP9 |
|  |  | hsa-miR-3184-5p\|microT-CDS | TCF7 |
|  |  | hsa-miR-3184-5p\|microT-CDS | ILK |
|  |  | hsa-miR-1233-5p\|microT-CDS | PIK3R5 |
|  |  | hsa-miR-6800-5p\|microT-CDS | TCF7L2 |
|  |  | hsa-miR-6800-5p\|microT-CDS | EGF |
|  |  | hsa-miR-5100\|microT-CDS | CTNNA3 |
|  |  | hsa-miR-4675\|microT-CDS | APC |
|  |  | hsa-miR-4675\|microT-CDS | MAPK1 |
|  |  | hsa-miR-5100\|Tarbase | CTNNB1 |
|  |  | hsa-miR-1290\|Tarbase | KRAS |
|  |  | hsa-miR-1290\|Tarbase | AKT2 |
|  |  | hsa-miR-6784-5p\|microT-CDS | APC2 |


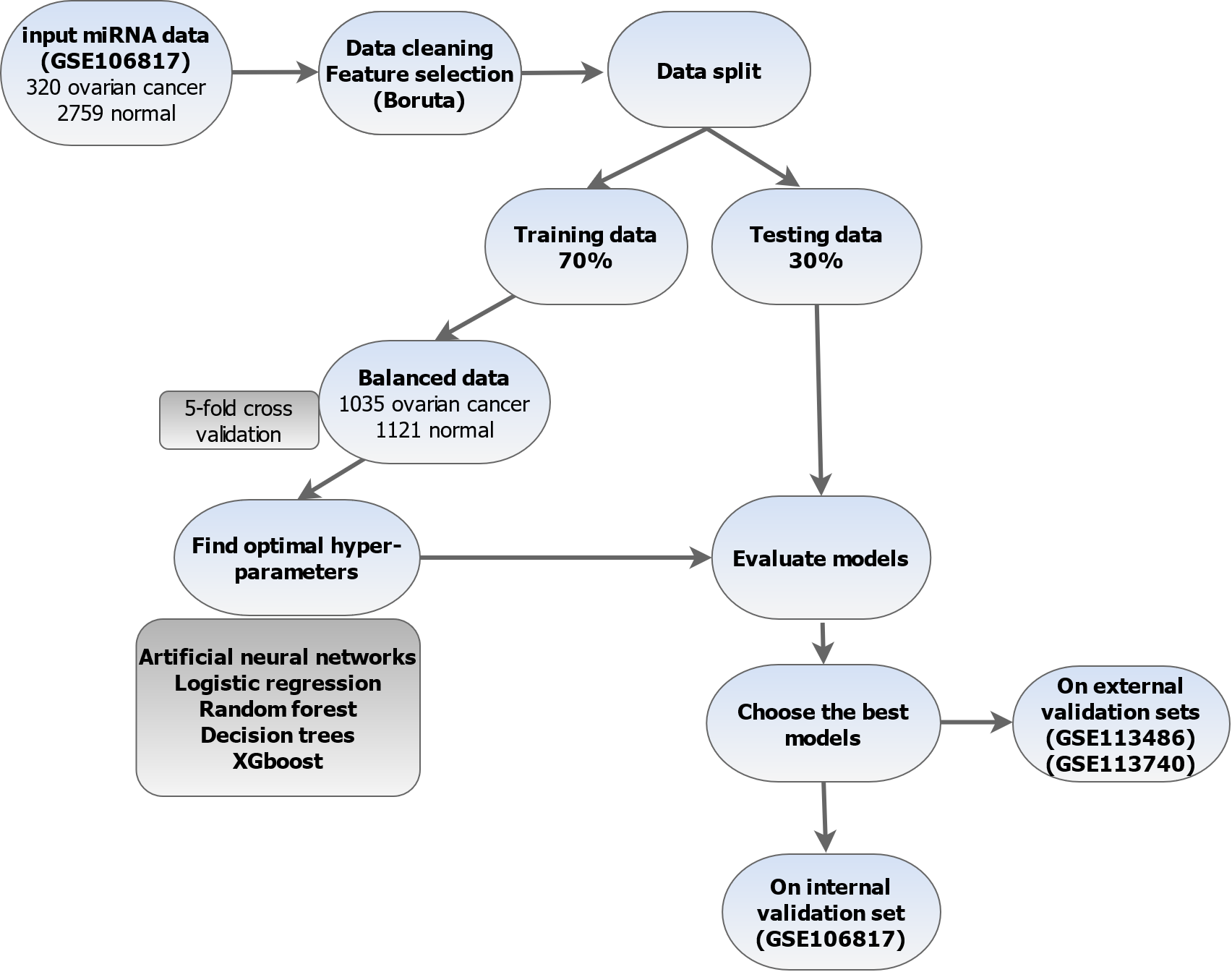


Supplementary Figure 1. flowchart of the research procedure.


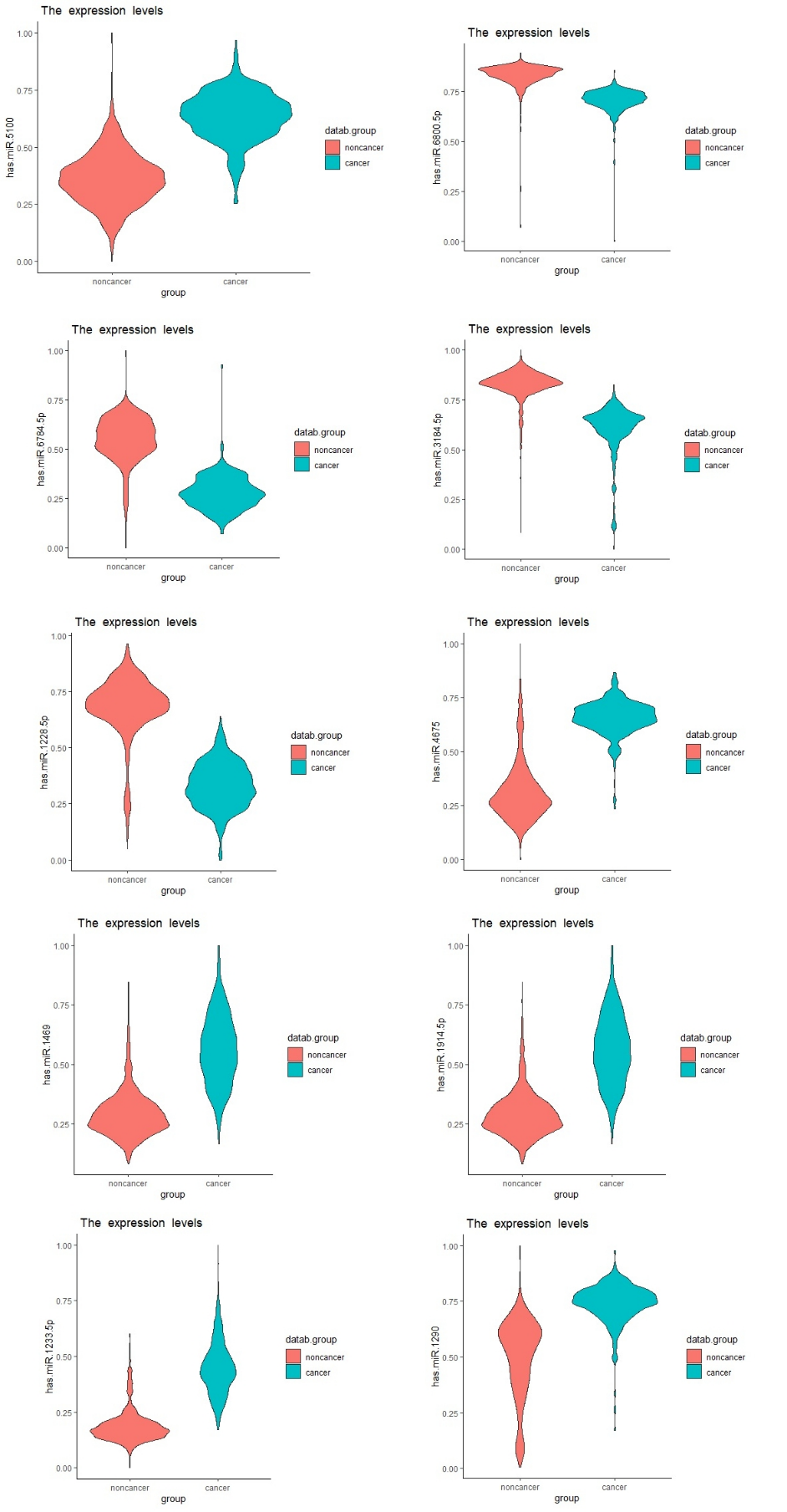


Supplementary Figure 2. Boxplot of each miRNA in GSE106817.


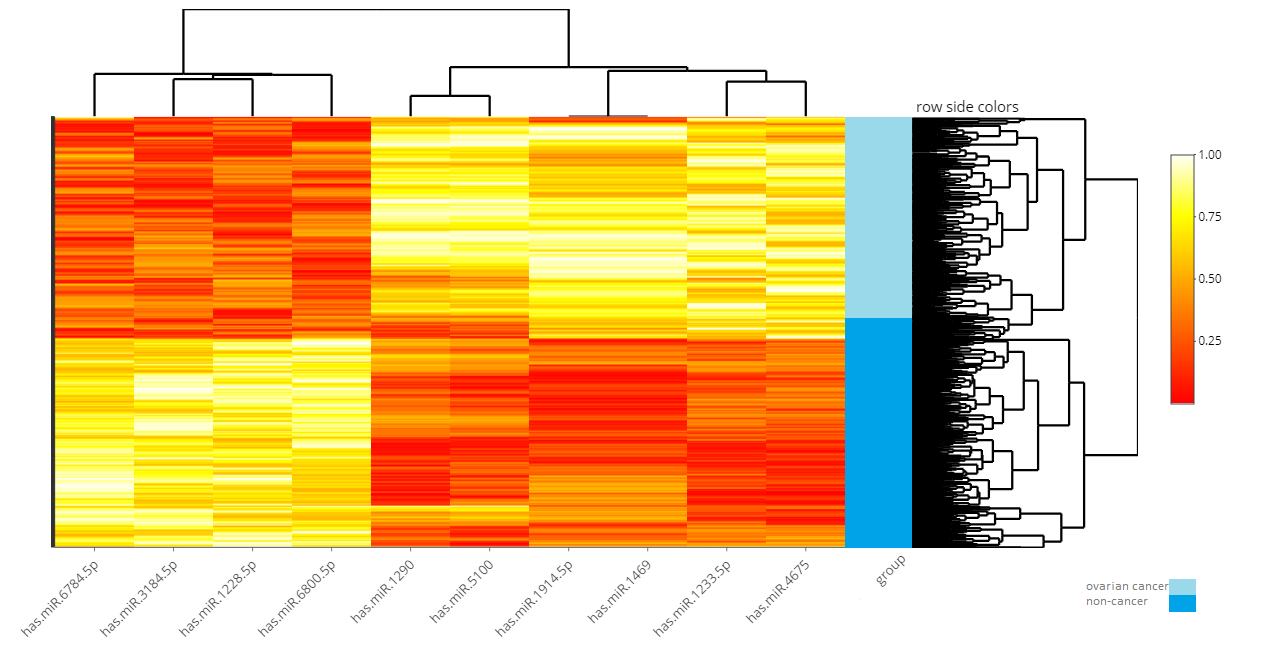


Supplementary Figure 3. Hierarchical cluster and heatmap of the expression of 10 miRNAs in GSE106817


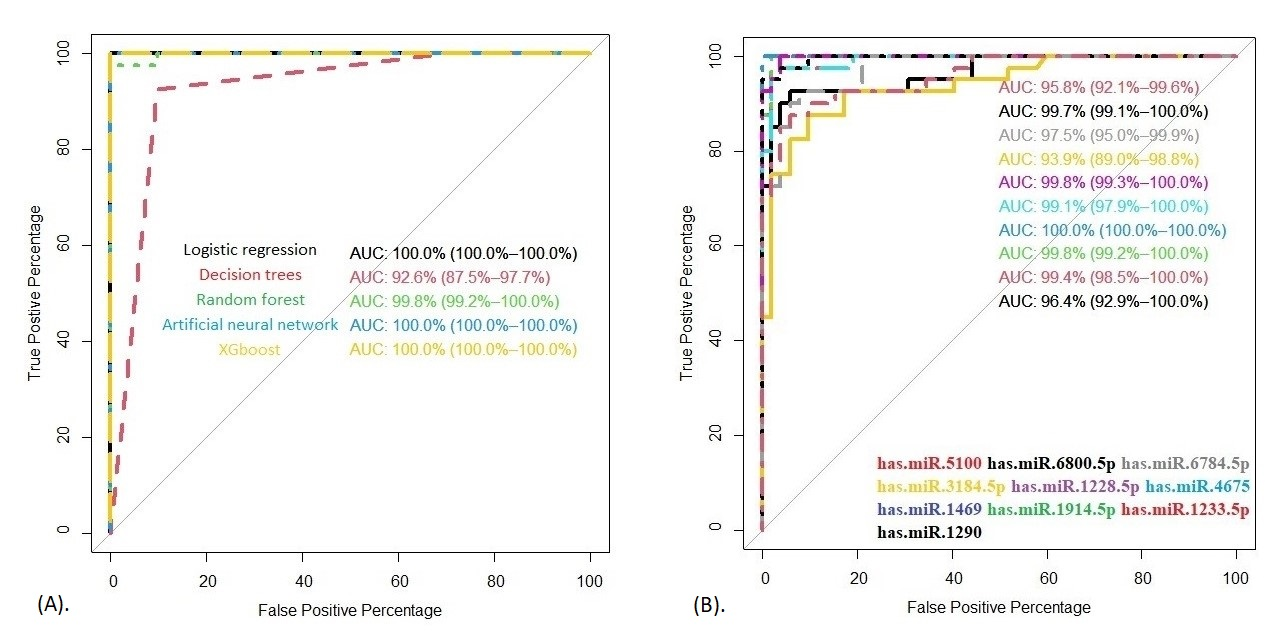


Supplementary Figure4. (A) ROC curve for proposed models of 10 candidate miRNAs in GSE113486. (B) ROC curve of each selected miRNA in GSE113486.


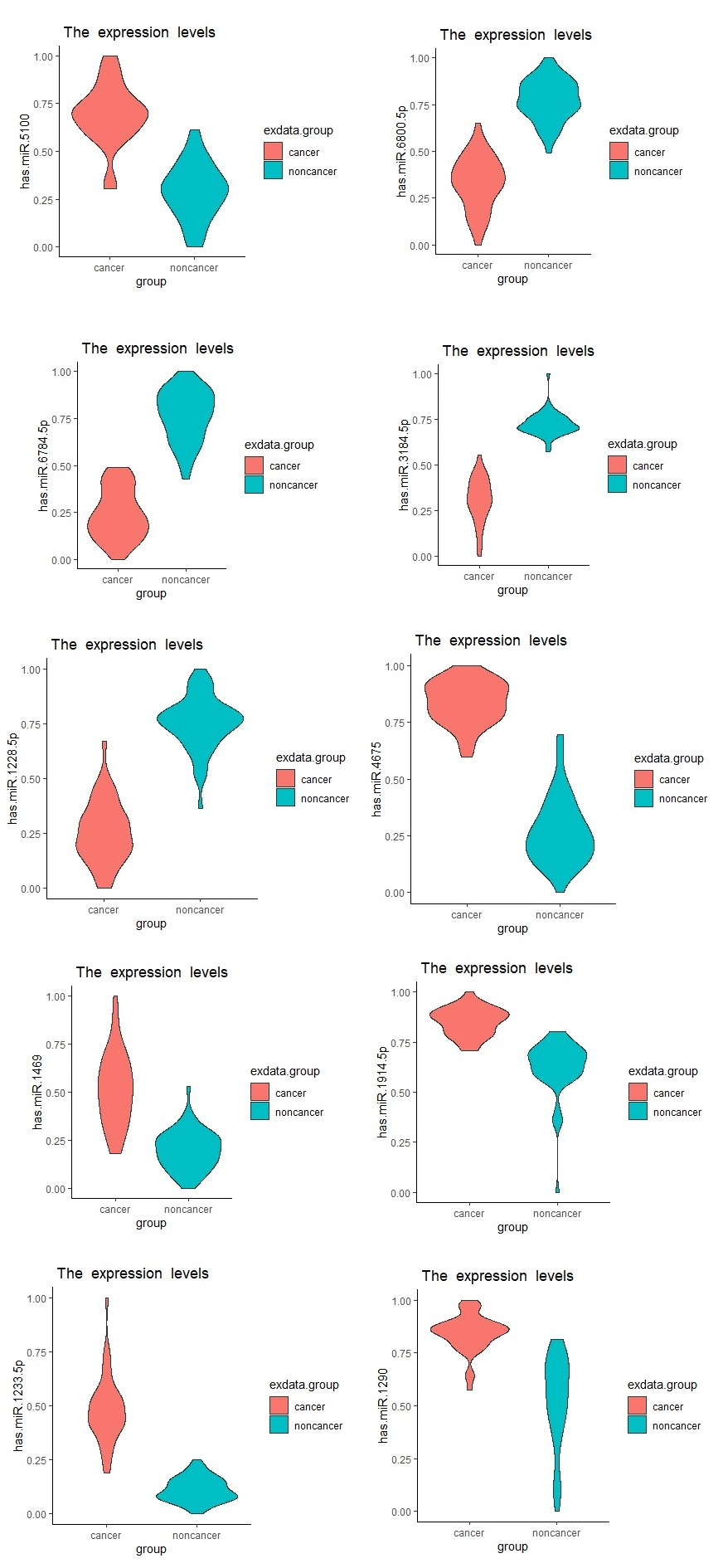


Supplementary Figure 5. boxplots of each miRNAs in GSE113486.


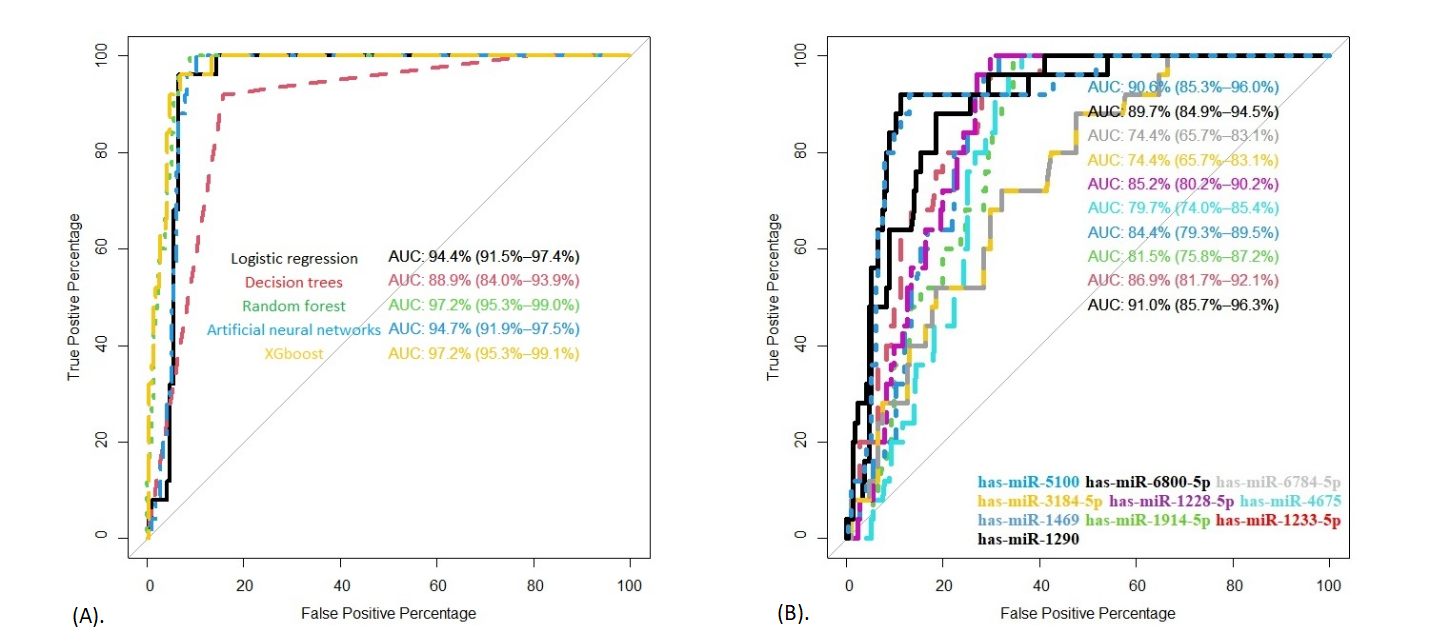


Supplementary Figure 6. (A) ROC curve for proposed models of 10 candidate miRNAs in GSE113740. (B) ROC curve of each selected miRNA in GSE113740.


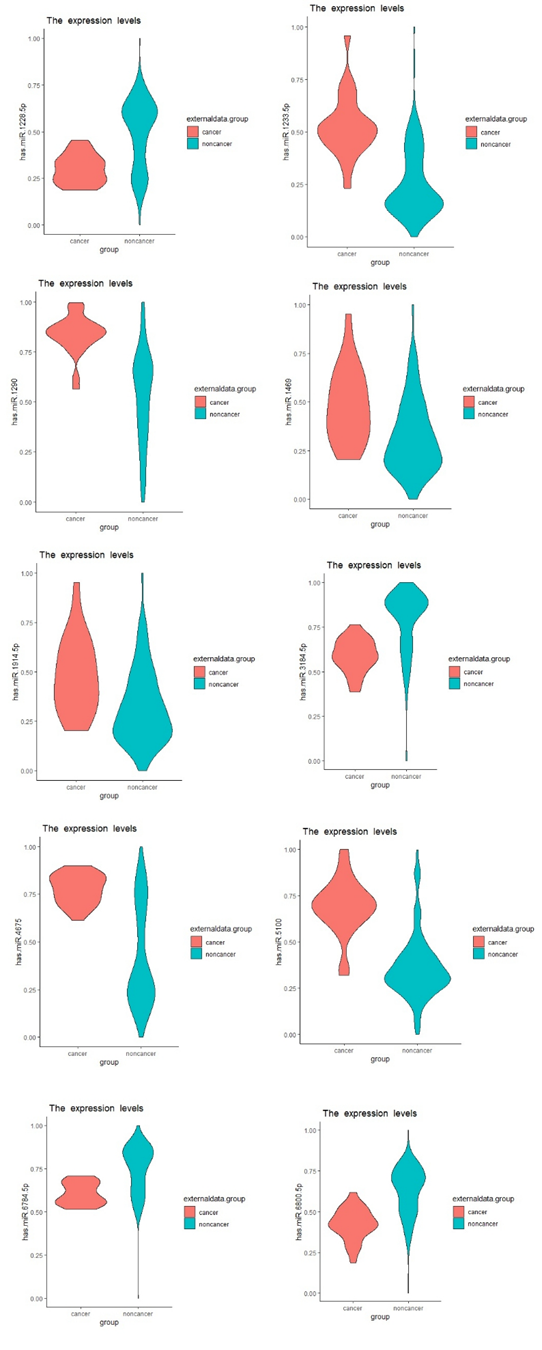


Supplementary Figure 7. Boxplot of each candidate miRNAs in GSE113740.


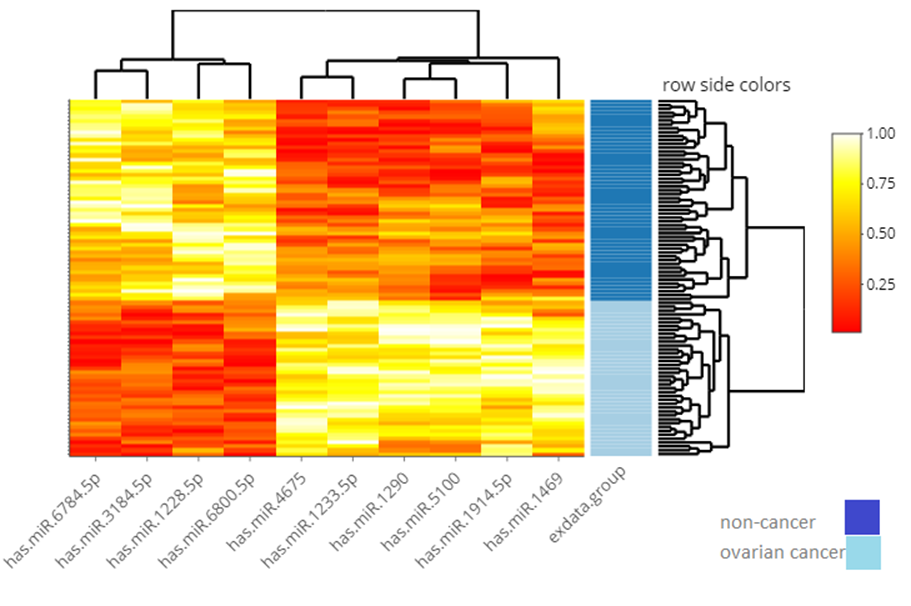


Supplementary Figure 8. Hierarchical cluster and heatmap of the expression of 10 miRNAs in GSE113486.


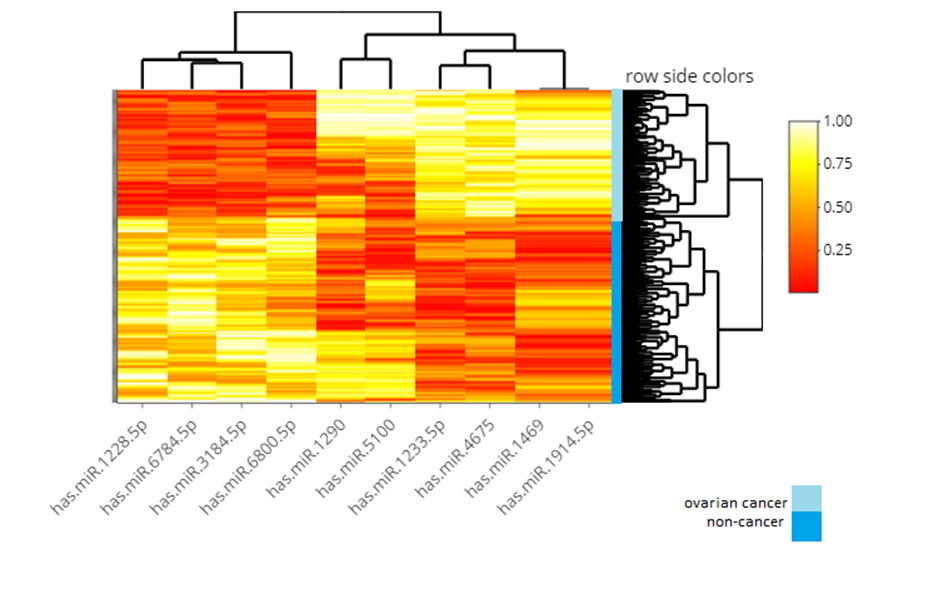


Supplementary Figure 9. Hierarchical cluster and heatmap of the expression of 10 miRNAs in GSE113740.

1. Guz M, Jeleniewicz W, Cybulski M. An Insight into miR-1290: An Oncogenic miRNA with Diagnostic Potential. *Int J Mol Sci.* (2022) 23(3):1234. doi: 10.3390/ijms23031234.

2. Ghafouri-Fard S, Khoshbakht T, Hussen BM, Taheri M, Samadian M. A Review on the Role of miR-1290 in Cell Proliferation, Apoptosis and Invasion. *Front Mol Biosci.* (2021) 8:763338. doi: 10.3389/fmolb.2021.763338.

3. Lai X, Cheng H. LncRNA colon cancer-associated transcript 1 (CCAT1) promotes proliferation and metastasis of ovarian cancer via miR-1290. *Eur Rev Med Pharmacol Sci.* (2018) 22(2):322-328. doi: 10.26355/eurrev_201801_14175.

4. Liu S, Qu D, Li W, He C, Li S, Wu G, et al. miR‑647 and miR‑1914 promote cancer progression equivalently by downregulating nuclear factor IX in colorectal cancer. *Mol Med Rep.* (2017) 16(6):8189-8199. doi: 10.3892/mmr.2017.7675.

5. Yamamura J, Ma S, Jia H, Kato H. Activating transcription factor 4-dependent hsa-miR-663a transcription mediates mTORC1/p70S6K1 signaling underleucine deprivation. *Front Nutr.* (2022) 9:965771. doi: 10.3389/fnut.2022.965771

6. Rajarajan D, Selvarajan S, Charan Raja MR, Kar Mahapatra S, Kasiappan R. Genome-wide analysis reveals miR-3184-5p and miR-181c-3p as a critical regulator for adipocytes-associated breast cancer. *J Cell Physiol.* (2019) 234(10):17959-17974. doi: 10.1002/jcp.28428.

7. Li D, Li Z, YanFei W, Wang Y, Shi J, Liu C, et al. LncRNA FOXP4‐AS promotes the progression of non‐small cell lung cancer by regulating the miR‐3184‐5p/EIF5A axis. *J Tissue Eng Regen Med.* (2022) 16(4):335-345. doi: 10.1002/term.3275.

8. Zhang HM, Li H, Wang GX, Wang J, Xiang Y, Huang Y, et al. MKL1/miR-5100/CAAP1 loop regulates autophagy and apoptosis in gastric cancer cells. *Neoplasia*. (2020) 22(5):220-230. doi: 10.1016/j.neo.2020.03.001.

9. Zhong D, Wu C, Xu D, Bai J, Wang Q, Zeng X. Plasma-Derived Exosomal hsa-miR-4488 and hsa-miR-1228-5p: Novel Biomarkers for Dermatomyositis-Associated Interstitial Lung Disease with Anti-Melanoma Differentiation-Associated Protein 5 Antibody-Positive Subset. *Biomed Res Int.* (2021) 2021:6676107. doi: 10.1155/2021/6676107.

10. Chen D, Ma W, Ke Z, Xie F. CircRNA hsa_circ_100395 regulates miR-1228/TCF21 pathway to inhibit lung cancer progression. *Cell Cycle*. (2018) 17(16):2080-2090. doi: 10.1080/15384101.2018.1515553.
